# Compensatory Evolution of *pbp* Mutations Restores the Fitness Cost Imposed by β-Lactam Resistance in *Streptococcus pneumoniae*

**DOI:** 10.1371/journal.ppat.1002000

**Published:** 2011-02-17

**Authors:** Andrea G. Albarracín Orio, Germán E. Piñas, Paulo R. Cortes, Melina B. Cian, José Echenique

**Affiliations:** Departamento de Bioquímica Clínica - CIBICI (CONICET), Facultad de Ciencias Químicas, Universidad Nacional de Córdoba, Córdoba, Argentina; Emory University, United States of America

## Abstract

The prevalence of antibiotic resistance genes in pathogenic bacteria is a major challenge to treating many infectious diseases. The spread of these genes is driven by the strong selection imposed by the use of antibacterial drugs. However, in the absence of drug selection, antibiotic resistance genes impose a fitness cost, which can be ameliorated by compensatory mutations. In *Streptococcus pneumoniae*, β-lactam resistance is caused by mutations in three penicillin-binding proteins, PBP1a, PBP2x, and PBP2b, all of which are implicated in cell wall synthesis and the cell division cycle. We found that the fitness cost and cell division defects conferred by *pbp2b* mutations (as determined by fitness competitive assays in vitro and in vivo and fluorescence microscopy) were fully compensated by the acquisition of *pbp2x* and *pbp1a* mutations, apparently by means of an increased stability and a consequent mislocalization of these protein mutants. Thus, these compensatory combinations of *pbp* mutant alleles resulted in an increase in the level and spectrum of β-lactam resistance. This report describes a direct correlation between antibiotic resistance increase and fitness cost compensation, both caused by the same gene mutations acquired by horizontal transfer. The clinical origin of the *pbp* mutations suggests that this intergenic compensatory process is involved in the persistence of β-lactam resistance among circulating strains. We propose that this compensatory mechanism is relevant for β-lactam resistance evolution in *Streptococcus pneumoniae*.

## Author Summary

For many years, pneumococcal infections have been usually treated with β-lactams. However, the rapid emergence of β-lactam resistance has complicated the antimicrobial treatment of these infections in the last two decades. The emergence and stability of antibiotic resistance is a complex biological process driven by different factors, such as the volume of antibiotic used. Furthermore, many studies on the effect of a reduction in β-lactam consumption have reported a sustained resistance level to *S. pneumoniae*, suggesting that other factors contribute to the persistence of β-lactam resistance. By horizontal gene transfer, *S. pneumoniae* is able to acquire genes from resistant strains or the commensal streptococci, which confer β-lactam resistance. Here, we show that when certain resistance genes are acquired individually, an important cost results in the bacterial fitness. However, some clinical strains which have acquired genes that increase β-lactam resistance can also compensate the fitness cost imposed by this resistance, thereby producing a selective advantage and raising the potential spreading of β-lactam resistance. We suggest that *pbp1a* and *pbp2x* mutant alleles are acquired for their compensatory effect on fitness in addition to their contribution in developing higher β-lactam resistance levels, and that this process may occur even in the absence of antibiotics.

## Introduction

*Streptococcus pneumoniae* is the causal agent of human infections such as otitis, pneumonia and meningitis, which particularly affect pediatric patients. Penicillin and other β-lactams (βLs) are the treatments of choice for any pneumococcal infection. However, β-lactam resistance has been growing steadily since the first clinical resistant isolate was reported in the 1960s, with its dissemination greatly threatening the clinical efficacy of these compounds [1]. βLs act by binding to the active sites of PBPs, which are involved in peptidoglycan synthesis, thus altering the normal cell wall formation and inducing cell death by lysis. Specifically, PBPs catalyze the last two stages of peptidoglycan biosynthesis, namely transglycosylation and transpeptidation. Its ability to uptake exogenous DNA by induction of competence allows *S. pneumoniae* to acquire antibiotic resistance mutations. β-lactam resistance results from the acquisition of mutations within the *pbp2b*, *pbp2x*, and *pbp1a* genes, causing multiple amino acid changes in PBP2b, PBP2x, and PBP1a, which decrease the enzyme affinity for βLs [2]. Exogenous DNA containing *pbp* mutations can be provided by β-lactam resistant (βL^R^) strains of *S. pneumoniae* or by other *Streptococcus* species that cohabit the same niche, and is incorporated into the chromosome by homologous recombination [3].

The rise in βL resistance detected among circulating strains is due to their ability to acquire *pbp* genes, as mentioned above, in association with the wide therapeutic use of βL compounds, which could act as a selective pressure [4]. In the absence of antibiotic therapy, bacterial antibiotic resistance genes commonly result in reduced fitness. Nevertheless, by acquiring intra or intergenic compensatory mutations, certain clinical and laboratory strains are able to restore this fitness cost [5], [6]. Presumably, this compensation allows these strains to compete with susceptible strains in their natural niches. In *S. pneumoniae*, ciprofloxacin-resistant isolates have a low prevalence due to a fitness cost imposed by point mutations in the genes that encode DNA polymerase and DNA gyrase [7], [8]. A putative relationship has also been suggested between βL resistance and the loss of virulence in pneumococcal clinical strains [9], [10]. Likewise, Rieux and coworkers [11] described a loss of virulence in single and double isogenic βL^R^ mutants, obtained by the transformation of *pbp2x* and *pbp2b* mutations amplified from βL^R^ clinical isolates. Related to this, Trzcinski and coworkers, studying clinical isolates [12], reported an increase in fitness cost when βL resistance was conferred by the *pbp2b pbp2x pbp1a* mutations acquired by sequential transformation events.

In this work, we studied the clinical βL^R^ strains belonging to a new serotype-14 variant of the Spain^9V^-3 international clone. This variant was isolated in Argentina and is indistinguishable by MLST and sequence analysis of the *cps* genes from those isolated in Baltimore, USA [13]. We demonstrated that the sequential acquisition of *pbp* mutations to develop βL resistance is closely associated to an intergenic compensatory process, which can restores the fitness cost imposed by *pbp2b* mutations and may favor the persistence and spread of βL resistance in *S. pneumoniae*.

## Results

### Fitness cost of β-lactam resistance in *S. pneumoniae*

The fitness cost imposed by *pbp2b*, *pbp2x* and *pbp1a* mutations that confer βL resistance in *S. pneumoniae* was evaluated. These *pbp* alleles were obtained from βL^R^ clinical strains that belonged to a new serotype-14 variant of the Spain^9V^-3 international clone, identified and characterized in our laboratory [13]. To generate strains bearing alleles of *pbp2b*, *pbp2x*, and *pbp1a* from clinical strains that conferred resistance, we used PCR to amplify the DNA region that encodes the transpeptidase domain of each mutated *pbp* gene (in *pbp1a*, the glycosyltransferase domain was also included; Fig. S1 in Supporting Information S1), where the βL resistance mutations are commonly localized. These PCR products containing the *pbp* mutations were introduced by transformation into the βL-susceptible laboratory strain Cp1015, and βL^R^ mutants were selected by growth on agar plates supplemented with piperacillin for *pbp2b*, or with cefotaxime for *pbp2x* and *pbp1a* (Table S1 in Supporting Information S2). The *pbp* mutations were integrated into the chromosome by homologous recombination.

Although no growth alterations for single *pbp2x* or *pbp1a* mutants were found, which presented similar growth curves to that of Cp1015, *pbp2b* mutants revealed an uncommonly prolonged lag-phase (Fig. S2 in Supporting Information S1). Then, due to the fact that clinical strains (from which *pbp* genes were amplified to generate single *pbp* mutants) grew as well as the wild-type strain (data not shown), we decided to investigate whether the fitness cost resulting from *pbp2b* mutations was compensated for by associating the *pbp2x* and *pbp1a* genes amplified from the same βL^R^ strain donor of *pbp2b*. For this purpose, double and triple *pbp* mutants were obtained by sequential transformation of single and double *pbp* mutants (in that order), and the βL^R^ mutant selection was made by using the MIC (Minimal Inhibitory Concentration) increment produced by the acquisition of different *pbp* mutations (Table 1). For example, a 14-fold increase occurred in the piperacillin MIC level for the *pbp2b* mutant; a 10-fold increase was found in the cefotaxime MIC level for the *pbp2x or pbp1a* mutants (single or double *pbp* mutants), and a 14-fold increase resulted in the cefotaxime MIC level for triple *pbp* mutants. The individual and double *pbp* mutants displayed a transformation rate higher than 10^−2^/µg of DNA, similar to the wild-type strain. Natural competence was also assayed, but no alterations in spontaneous transformability were detected (Fig. S3 in Supporting Information S1). We observed a partial restitution of the growth curve for the double *pbp2b pbp2x* and *pbp2b pbp1a* mutants, while a clear restoration was found for the triple *pbp* mutant (Fig. S2 in Supporting Information S1). Interestingly, all seven *pbp2b* mutants constructed by transformation with different mutated *pbp2b* genes obtained from other non-susceptible clinical isolates (Cba strains, Table S1 in Supporting Information S2) and the Spain^9V^-3 ATCC strain displayed similar growth alterations (data not shown). These *pbp2b* alleles were sequenced, and a comparison between them revealed that the DNA sequences obtained from Cba-19, Cba-28, Cba-52, Cba-62 and ATCC 700671 (clone Spain^9V^-3) were identical (Fig. S4 in Supporting Information S1). It was also found that 5 amino acids were conserved in the transpeptidase domain for all the *pbp2b* sequences (P417, E443, I460, G481 and A494) obtained from the different βL^R^ clinical strains.

**Table 1:** Relative competitive fitness of *Streptococcus pneumoniae pbp* mutants.

*pbp* mutants	Relative fitness in vitroa,b			MIC (µg/ml)		
	Mean	SD	(95% CI)	PIP	CEF	PEN
**strain CP1015**				0.015	0.015	0.02
*pbp2b^19^*	0.77	0.02	0.74–0.80	0.2	0.015	0.02
*pbp1a^19^*	1.10	0.06	1.04–1.18	0.015	0.15	0.02
*pbp2x^19^*	0.98	0.05	0.90–1.06	0.015	0.15	0.02
*pbp2b^19^*, *pbp1a^19^*	1.02	0.04	0.96–1.08	0.2	0.15	0.15
*pbp2b^19^*, *pbp2x^19^*	1.06	0.04	0.99–1.12	0.2	0.15	0.15
*pbp2b^19^ pbp2x^19^ pbp1a^19^*	0.95	0.05	0.87–1.03	0.2	0.2	0.3
*pbp2b^28^*	0.65	0.02	0.62–0.68	0.2	0.015	0.02
*pbp1a^28^*	1.05	0.05	1.03–1.12	0.015	0.12	0.02
*pbp2x^28^*	0.97	0.04	0.90–1.03	0.015	0.15	0.02
*pbp2b^28^*, *pbp1a^28^*	1.03	0.04	0.97–1.09	0.2	0.12	0.02
*pbp2b^28^ pbp2x^28^*	0.86	0.01	0.84–0.87	0.2	0.15	0.15
*pbp2b^28^ pbp2x^28^ pbp1a^28^*	1.10	0.04	1.04–1.16	0.2	0.2	0.15
*pbp2b^9V3^*	0.65	0.04	0.59–0.71	0.18	0.015	0.02
*pbp2x^9V3^*	0.80	0.04	0.74–0.86	0.015	0.12	0.02
*pbp2b^9V3^*, *pbp2x^9V3^*	1.08	0.04	1.02–1.14	0.18	0.12	0.2
*pbp2b^9V3^*, *pbp2x^9V3^*, *pbp1a^9V3^*	1.10	0.05	1.02–1.18	0.18	0.12	0.3
*pbp2b^28^*, *pbp1a^28^/A2*	0,96	0.05	0,83–1.08	0.2	0.12	0.1
*pbp2b^28^ pbp1a^28^/A3*	0,70	0.05	0,58–0.82	0.2	0.15	0.04
*pbp2b^28^ pbp2x^28^/X4*	0,64	0.05	0,52–0.76	0.2	0.15	0.1
**strain D39**						
D39 *pbp2b^9V3^*	0.61	0.01	0.59–0.63	0.18	0.015	0.02
D39 *pbp2b^9V3^ pbp2x^19^*	0.75	0.05	0.67–0.83	0.18	0.15	0.15
D39 *pbp2b^9V3^ pbp2x^19^ pbp1a^19^*	1.10	0.02	1.07–1.13	0.18	0.2	0.15
						

*pbp* mutants	Relative fitness in vivo^b^:			MIC (µg/ml)		
	Mean	SD	(95% CI)	PIP	CEF	PEN
D39 *pbp2b^9V3^*	0.27	0.09	0.19–0.35	0.18	0.015	0.02
D39 *pbp2b^9V3^ pbp2x^19^*	0.80	0.1	0.70–0.89	0.18	0.15	0.15
D39 *pbp2b^9V3^ pbp2x^19^pbp1a^19^*	1.14	0.2	0.95–1.33	0.18	0.2	0.15

a Relative competitive fitness is given by the ratio of the number of generations of the resistant and susceptible CP1015 strains.

b Relative fitness in vitro and in vivo was determined as described in Material and Methods.

References: SD, standard deviation; CI, confidence intervals; 9V3, Spain^9V^-3 international clone; CEF, cefotaxime; PIP, piperacillin; PEN, penicillin.

These results suggest that these substitutions could be responsible for the fitness cost detected in the *pbp2b* mutants. The *pbp2b* sequences of mutants constructed in the Cp1015 strain were identical to those obtained from the clinical strains, with each *pbp2b* sequence being coincident with the original *pbp2b* from the clinical strains used to transform the wild-type strain. Hereafter, the strain reference will be expressed as superscripts in the *pbp* mutations; for example, the *pbp2b* mutation from strain Cba-28 that was transformed into the Cp1015 strain will be indicated as *pbp2b^28^*.

### Fitness cost compensation between *pbp* mutations associated to β-lactam resistance

**Figure 1:**
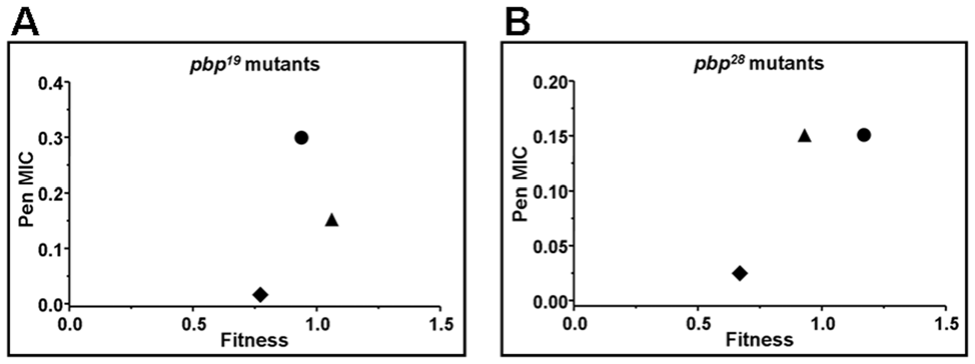
Correlation plot between the fitness value of *pbp* mutants, obtained by using *pbp* genes from Cba-19 and Cba-28 clinical strains, and the penicillin MIC increase found (µg/ml). A) Correlation for Cp1015 *pbp^19^* mutants, Cp1015 *pbp2b^19^* (diamonds), Cp1015 *pbp2b^19^ pbp2x^19^* (triangles), and Cp1015 *pbp2b^19^ pbp1a^19^ pbp2x^19^* (circles). B) Correlation for Cp1015 *pbp^28^* mutants, Cp1015 *pbp2b^28^* (diamonds), Cp1015 *pbp2b^28^pbp2x^28^* (triangles), and Cp1015 *pbp2b^28^ pbp2x^28^ pbp1a^28^* (circles).

All findings about compensation of growth alterations were similar to the results obtained by competitive fitness assays in vitro, when comparing the βL-susceptible Cp1015 strain with isogenic *pbp* mutants. The compensatory effect among *pbp* mutations was evident not only in triple, but also in *pbp2b^19^ pbp1a^19^*, *pbp2b^19^ pbp2x^19^*, *pbp2b^28^ pbp1a^28^*, and *pbp2b^9V3^ pbp2x^9V3^* double mutants, recovering their values of relative fitness of 0.95–1.10 in the case of the triple *pbp* mutants (Table 1). We also observed that single *pbp1a* mutations obtained from Cba-19 and Cba-28 increased fitness in the wild-type strains (Table 1), and therefore should be considered as increasing-fitness mutations. Interestingly, we found a direct correlation between fitness compensation and an increase in the spectrum and level of βL resistance (Fig. 1; Table 1).

The acquisition of *pbp2x* and *pbp1a* mutations from clinical βL^R^ strains other than Cba-28 was also able to compensate the fitness cost found in *pbp2b^28^* mutants. We observed that fitness cost was ameliorated in 7 of the 8 double *pbp2b^28^ pbp2x* mutants analyzed, which were constructed by transforming *pbp2x* genes from donor βL^R^ strains different from Cba-28 into the *pbp2b^28^* mutant (Table S2 in Supporting Information S2). When the transpeptidase regions of *pbp2x* sequences from the different mutants constructed in Cp1015 were compared, we observed that the *pbp2x* sequences in 4 mutants (*pbp2b^28^ pbp2x^12^*, *pbp2b^28^ pbp2x^52^*, *pbp2b^28^ pbp2x^54^* and *pbp2b^28^ pbp2x^62^*) presented 10 conserved substitutions compared with Cp1015, with the exception of *pbp2x^52^*, which showed D278 in place of N278 (Fig. S5 in Supporting Information S1). Coincidently, *pbp2x^52^* was the only one of these 4 *pbp2x* alleles that could not ameliorate the fitness cost of *pbp2b^28^* (Table S2 in Supporting Information S2). This result suggests that the N278 residue might be involved in the compensatory mechanism of fitness, at least for the set of mutations present in *pbp2x^12^*, *pbp2x^54^* and *pbp2x^62^*. Each *pbp2x* sequence used to transform Cp1015 was identical to the original *pbp2x* obtained from clinical strains (data not shown).

To corroborate the fitness compensation of *pbp* mutations observed in vitro, we also evaluated the relative fitness in vivo of single, double and triple *pbp* mutants using a model of intraperitoneal infection in C57BL/6 mice. Due to the fact that Cp1015 is an avirulent strain, we transferred the *pbp* mutations into the D39 strain, a virulent capsulated strain, which is able to infect C57BL/6 mice [14]. Single *pbp* mutants were obtained in D39 as described for Cp1015. Curiously, however, only crossed double and triple *pbp* mutants were recovered by transformation of the *pbp2x* and *pbp1a* genes from the Cba-19 strain into the D39 *pbp2b* ^9V3^ mutant (Table 1), indicating that the βL resistance conferred by these *pbp* mutations was dependent on the genetic background of the recipient strain. In agreement with the data obtained by assays in vitro, we observed a similar compensatory phenomenon for the D39 *pbp* mutants in a mice model, with values of relative fitness of 0.27 for *pbp2b^9V3^*, 0.80 for *pbp2b^9V3^ pbp2x^19^*, and 1.14 for the triple *pbp2b^9V3^ pbp2x^19^ pbp1a^19^* mutant being obtained (Table 1).

βL resistance is complex and it has been reported that is conferred by mosaic DNA fragments containing several mutations in the transpeptidase domain, which decrease the binding of βLs. With the purpose of identifying the amino acid substitutions that could be involved in the fitness compensation mechanism described, sub-parts of the *pbp2x* and *pbp1a* genes from the βL^R^ Cba-28 strain were amplified by PCR (see schemes shown in Figs. S6 and S7 in Supporting Information S1), and PCR products were independently transformed into the *pbp2b^28^* mutant, with the transformants being selected by cefotaxime resistance. We showed that fragments A2, A3 (both amplified from *pbp1a^28^*) and X4 (amplified from *pbp2x^28^*) conferred resistance, but only A2 could also compensate the fitness alterations (Table S3 in Supporting Information S2), reaching the same level as that obtained when *pbp2b^28^* was transformed with the *pbp1a^28^* fragment containing the glycosyltransferase and transpeptidase domains (Table 1). We analyzed the amino acid sequences deduced from the DNA sequences of A2 and A3, and found four substitutions shared between both regions (Fig. S8 in Supporting Information S1), which are probably involved in βL resistance. Interestingly, the E285Q mutation, localized in the transpeptidase domain [15], was only present in A2 (from *pbp1a^28^*). We propose that the E285Q mutation may have contributed to the fitness compensation mechanism. The presence of all these mutations was found in the double mutants *pbp2b^28^ pbp1a^28^* and *pbp2b^28^ pbp2x^28^* constructed in the Cp1015 genetic background as well as in the clinical Cba-28 strain.

In contrast with our results, Trzcinski and coworkers [12] reported that the fitness cost of pneumococcal penicillin-resistant strains increased with the number of resistant *pbp* alleles acquired. On comparing the predicted amino acid sequences of the *pbp* alleles studied here with those obtained by Trzcinski and coworkers, we found clear differences between them, detecting 9, 11 and 12 substitutions in PBP2x, PBP1a and PBP2b, respectively (Figs. S9, S10 and S11 in Supporting Information S1). This suggests that the discrepancy found between these works may be due to specific *pbp* mutations.

### Cell division alterations associated to *pbp* mutations

**Figure 2:**
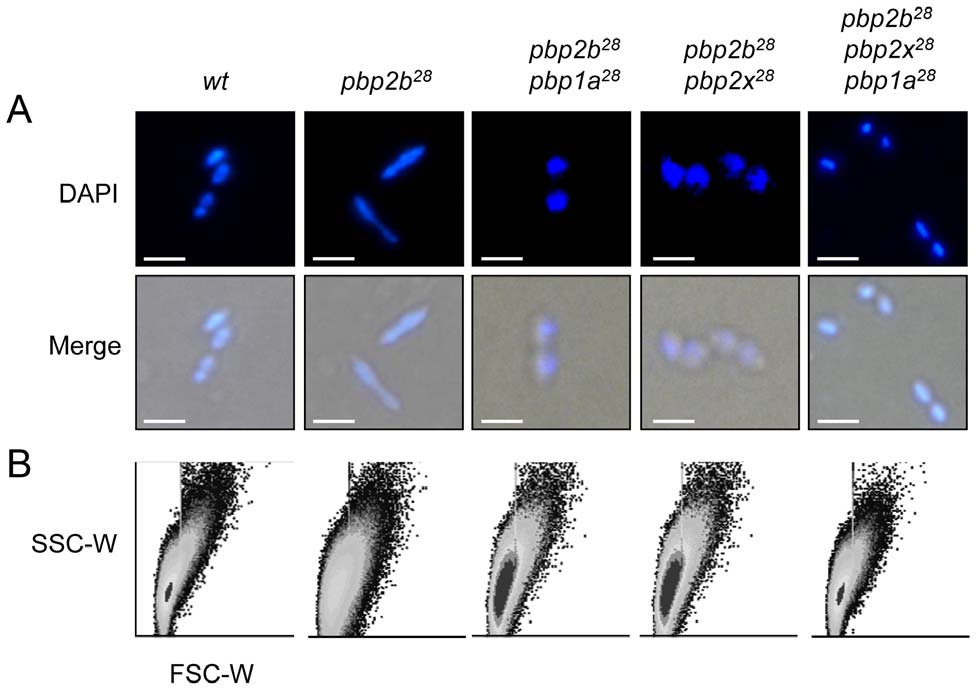
Effect of *pbp* mutations on cell morphology. Cells were prepared for microscopy and stained with DAPI as described in Materials and Methods (bar scale, 5 µm). (A) Merged phase-contrast and DAPI fluorescence image of the *pbp* mutants showing a bacillar morphology, contrasting with the typical coccoid shape of the CP1015 (*wt*) strain. (B) Cell shape modifications of the cell populations detected by flow cytometry analysis. Exponentially growing bacterial cells were injected into a flow cytometer and the results were analyzed with the WinMDI software. To compare populations, the data for each strain were plotted on a two-dimensional graph (*x*-axis, forward scatter; *y*-axis, side scatter).

It is known that PBPs are responsible for peptidoglycan synthesis. These proteins form the main component of the cell wall, and are involved in longitudinal wall growth and the cell division process. Morlot and coworkers [16] have proposed certain functions for PBP2b, PBP2a and PBP1a in *S. pneumoniae*, but the specific roles of PBPs in the growth and division phases are not yet well understood in bacteria. PBPs are also recognized as the targets for βLs, the most frequently used antimicrobials for treating bacterial infections. Therefore, considering that the interaction between drug resistance and the cell division processes remains unclear [4], we decided to investigate this connection. First, we examined the effects of *pbp* mutations on *S. pneumoniae* cell morphology, and then the putative cell division alterations. Microscopic examinations of single *pbp1a^28^* and *pbp2x^28^* mutants in Cp1015 strain did not display any morphological alterations, whereas the *pbp2b^28^* mutant (Fig. 2A) and all 10 strains bearing *pbp2b* mutations from clinical strains showed a rod shape (data not shown). This atypical phenotype was also found for *pbp2b* mutants with a different genetic background to that of Cp1015, such as the R6 and D39 laboratory strains (data not shown).

**Figure 3:**
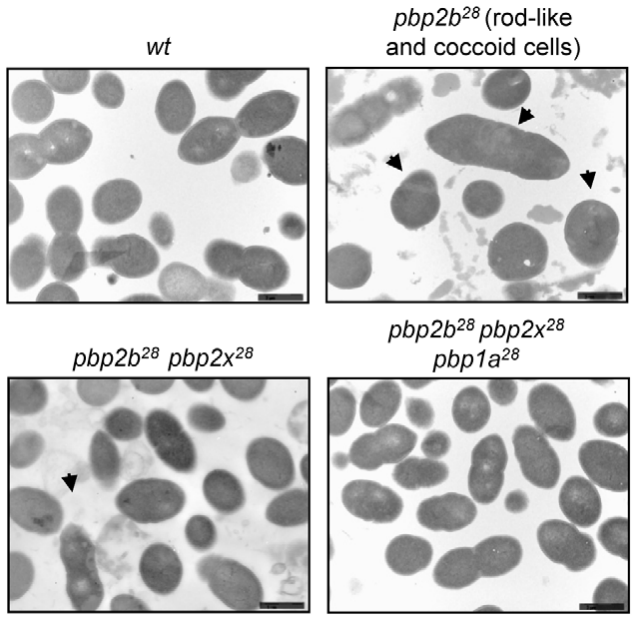
Electron microscopy analysis of cell morphology of *pbp* mutants. Microphotographs of the wild-type strain (Cp1015) revealed the typical morphology of *S. pneumoniae*, with a correct septum placement, division and symmetry of daughter cells. In the *pbp2b^28^* mutant, two subpopulations were identified, namely rod-like and coccoid cells. The rod-like shaped cells showed multiple septa with incorrect formation and placement. The coccoid-shape cells exhibited multiple septa and intracellular accumulation of the cell wall, with the atypical septum localization resulting in irregular cell divisions and asymmetrical daughter cells (see arrows). The *pbp2b^28^ pbp2x^28^* mutant partially restored the coccoid morphology, but conserved some cell alterations similar to those of the single *pbp2b^28^* mutant. The triple *pbp* mutant showed a similar morphology to *wt* cells. Additional microphotographs are shown in Figs. S12, S13, S14 and S15 in Supporting Information S1 (bar scale, 1.6 µm).

Curiously, 46% of *pbp2b^28^* cells had a rod-like shape (Fig. 2A), with the rest showing an apparent wild-type morphology. Electron microscopy revealed that the coccoid-shaped cells exhibited a variety of cell wall defects, including an abnormal septum position, atypical intracellular structures and frequent asymmetrical divisions (Figs. 3 and S13 in Supporting Information S1) compared with the wild-type strain (Figs. 3 and S12 in Supporting Information S1), with the rod-shaped cells exhibiting multiple septa (Figs. 3 and S13 in Supporting Information S1). Moreover, the double *pbp2b^28^ pbp2x^28^* mutant displayed a coccoid morphology but with atypical septum localizations and peptidoglycan accumulation (Figs. 3 and S14 in Supporting Information S1). However, the triple *pbp* mutants had wild-type cell morphology and showed no ultrastructural alterations (Figs. 3 and S15 in Supporting Information S1).

**Figure 4:**
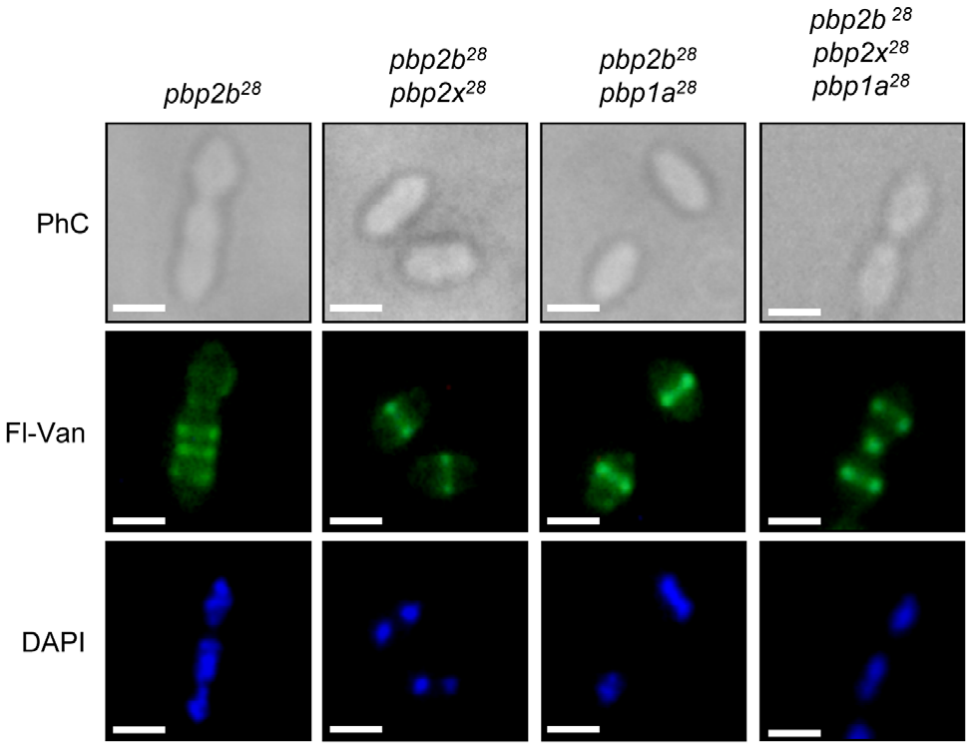
Septal localization in *pbp* mutants. Exponentially grown cells were stained with DAPI and fluorescent vancomycin (Fl-Van, which labels nascent peptidoglycan synthesis and strongly marks septal localization) and analysed by using an epifluorescence microscope (see Material and Methods). The *pbp2b* showed a septal accumulation in rod-like shaped cells, whereas the double (*pbp2b pbp2x* or *pbp2b pbp1a*) and the triple *pbp* mutants restored their septal localization. Representative images are shown from experiments that were repeated independently three times (bar scale, 1 µm).

When cells were stained with fluorescein-labeled vancomycin (Van-FL), which localizes to sites of nascent peptidoglycan synthesis and clearly marks the septum location in the wild-type strain [17] (Fig. S16 in Supporting Information S1), an abnormal septum pattern was revealed in rod-shaped cells of the *pbp2b^28^* mutants, suggesting a clear alteration in cell division (Fig. 4). However, this phenomenon was compensated in the double (*pbp2b^28^ pbp2x^28^* or *pbp2b^28^ pbp1a^28^*) and triple *pbp* mutants (Fig. 4). The morphological variation was confirmed by flow cytometry analysis, which allowed determining the population distribution of pneumococci by cell size. These assays showed a displacement favoring a larger cell size in the *pbp2b^28^* mutant, and the restoration of normal size in the triple *pbp* mutant (Fig. 2B). These results suggest that the septal alterations and the cellular enlargement found in the *pbp2b^28^* mutant could have been responsible for its growth retardation.

**Figure 5:**
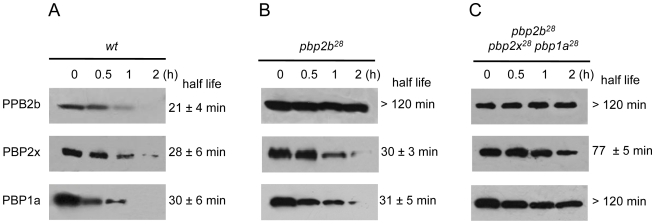
*S*tability in vivo of wild-type and mutant PBPs. Strains Cp1015 (Panel A), Cp1015 *pbp2b^28^* (Panel B) and Cp1015 *pbp2b^28^pbp2x^28^ pbp1a^28^* (Panel C) expressing HA-tagged PBPs were grown in Todd Hewitt broth with 0.5% Yeast Extract to an OD_600 nm_ of 0.2 prior to the addition of kanamycin (500 µg/ml) to inhibit protein synthesis. At the time intervals indicated, aliquots were withdrawn and analyzed by SDS-PAGE followed by immunoblotting with an anti-HA monoclonal antibody (see Material and Methods).

To investigate the putative cause of these effects on cell morphology and fitness, we analyzed the stability of the proteins encoded by these *pbp* mutated genes using immunoblotting and inhibiting the protein synthesis by the addition of kanamycin. For these assays, we constructed C-terminal HA tagged PBPs to permit the detection of the proteins using an anti-HA monoclonal antibody. The gene constructs were inserted into the chromosome by insertion-duplication as described previously [18], and these genes were expressed as single copies under the control of their native promoters. These *pbp-HA* mutants showed the same phenotypes as the original *pbp* mutants, as well as the compensatory effects demonstrated in the double and triple *pbp* mutants (data not shown). We observed an increase in the half-life (>120 min) for PBP2b^28^ compared with the wild-type protein (21 min), not only in *pbp2b^28^* (Fig. 5A) but also in the triple *pbp* mutant (Fig. 5C). Interestingly, we also detected an increased half-life for PBP1a^28^ (>120 min) and PBP2x^28^ (77 min) in the triple *pbp* mutant compared with half-life of PBP1a (31 min) and PBP2x (29 min) displayed in the wild-type strain (Figs. 5B and 5C). Our hypothesis is that these stability changes in the PBP mutants could have been responsible for the fitness/morphological alterations in the *pbp2b* mutant and the compensatory effects in the triple *pbp* mutant, and this will be discussed later.

**Figure 6:**
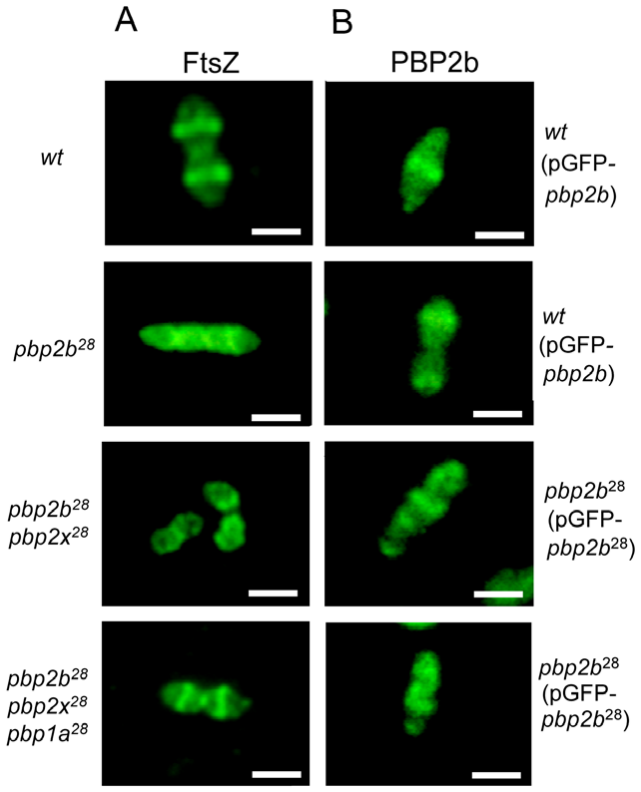
Protein localization in wild-type and *pbp* mutants by fluorescence microscopy. A) Localization of FtsZ. Microphotographs indicate the most common patterns of FtsZ localization through the cell cycle for *wt* and *pbp* mutants. Polyclonal antibody against FtsZ was used for its detection by immunofluorescence microscopy. In the Cp1015 (*wt*) strain, FtsZ is localized at the equatorial ring and septum, In Cp 1015 *pbp2b^28^*, the patterns suggest FtsZ delocalization and cell cycle disruption. The triple *pbp* mutant showed a wild-type FtsZ placement. B) Localization of PBP2b^28^-GFP. Microphotographs indicate the most common patterns found for PBP2b localization through the cell cycle in the *wt* and *pbp2b^28^* mutant. Representative images are shown from experiments that were repeated independently three times (bar scale, 1 µm).

**Figure 7:**
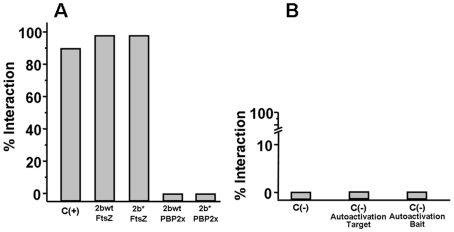
Bacterial two hybrid assay. The percentage of interaction is defined as the ratio between the number of cells co-transformed able to grow in 3-AT selective medium and the total number of cells obtained in medium without 3-AT. The plasmid pair pBT-LGF2/pTRG-Gal11 (BacterioMatch II, Stratagene) was used as a positive control. A) Interaction of PBP2b^wt^-FtsZ and PBP2b^28^-FtsZ B) As negative controls, the following plasmid pairs were used: pBT/pTRG carrying codifying sequences of non-interacting proteins, C (-); pBT-empty/pTRG-recombinant, to evaluate the two-hybrid system auto-activation for the target proteins (-)/Autoactivation Target; pBT-PBP2b/pTRG-empty, as non-specific transcription activation control mediated by PBP2b^wt^ or PBP2b^28^, C (-)/Autoactivation Bait.

We also investigated the putative cause of a morphological change in the *pbp2b^28^* mutant, and particularly in the cell division process. It was previously proposed that the life cycle of bacterial cells consists of repeated controlled enlargement, septum formation, and cell division [19]. In this scenario, FtsZ is an essential protein, which was postulated as the force generator that drives the cell division process, since the correct localization of all proteins involved in this mechanism is dependent on FtsZ [19], [20]. To evaluate the impact of *pbp* mutations on cell division, we determined the FtsZ localization by immunofluorescence microscopy, using a polyclonal antibody against FtsZ. Before septum formation, the FtsZ division ring has been described to be localized at the mid cell, as we also observed for the wild-type strain (Fig. 6A). However, an atypical FtsZ placement was found in *pbp2b* cells, with an apparent helical structure rather than the mid-cell localization found in wild-type cells. In the *pbp2b^28^ pbp2x^28^* mutants, although we observed a coccoid morphology, the FtsZ localization was still altered. In contrast, a total FtsZ placement restoration was found in the triple *pbp* mutants (Fig. 6A). The higher stability of PBP2b^28^ led us to speculate that this increased protein level could also cause a delocalization of this protein mutant. Therefore, we constructed the PBP2b-GFP and PBP2b^28^-GFP fusions, expressed ectopically from a multicopy plasmid, in order to study their localization in *S. pneumoniae* cells. We observed that PBP2b-GFP was localized equatorially in the wild-type strain as described previously [16]. However, PBP2b^28^-GFP revealed an atypical helical distribution, similar to FtsZ in the *pbp2b^28^* mutant (Fig. 6B), suggesting that PBP2b could interact with FtsZ. To investigate this, we analyzed this putative interaction by using a bacterial two-hybrid system (Bacteriomatch II, Stratagene), confirming this hypothesis by the detection of a positive interaction between PBP2b^28^ (or PBP2b) and FtsZ, but not between PBP2b^28^ (or PBP2b) and PBP2x. This served as a control of specificity in addition to the positive and negative controls included in this system (Fig. 7).

## Discussion

Mutations associated with antibiotic resistance are known to impose a fitness cost in several bacterial species. Nevertheless, this physiological cost can be ameliorated by compensatory mutations in the same altered gene or in others involved in the developed resistance mechanism, thereby maintaining the same resistance level [21]. This compensatory phenomenon can occur with or without selective pressure. However, under antibiotic exposure, only those mutations able to maintain the resistance level will be selected, thus permitting the survival and persistence of resistant strains [22].

In *S. pneumoniae*, individual *pbp2b* or *pbp2x* mutations are considered primary βL resistance determinants that confer low-level resistance, whereas *pbp1a* mutations are acquired later and are responsible for increasing this level. In this work, only *pbp2b* mutations were related to a significant fitness cost, indicating that these mutations were a disadvantage when competing with wild-type strains. This finding indicates that the *pbp2b* mutants were only able to survive under antibiotic pressure due to their resistance. However, in the absence of antibiotics, the only way for these mutants to compete with wild-type strains is by improving their fitness by acquisition of compensatory mutations.

In this work, one of the most important findings is that these compensatory mutations are also responsible for an increase in βL resistance. It is well known that the combination of *pbp* mutations that confers βL resistance is very complex, and that *pbp* alleles have DNA mosaics with mutations in the region that encodes the transpeptidase domain. These DNA mosaics may differ by over 20% from DNA sequences from sensitive *pbp* genes and from each other, resulting in changes in amino acid of more than 10% [23]. In this scenario with PBPs showing multiple mutations, it is very difficult to distinguish those involved in resistance development from the alterations caused by the natural evolution of the genes. Therefore, the contribution of each mutation is unclear, with the exception of a few conserved mutations in the transpeptidase domains of PBPs. In an attempt to identify the amino acids involved in the compensatory mechanism described here, different fragments of *pbp1a^28^* and *pbp2x^28^* were transformed into the *pbp2b^28^* mutant, and the resistance and fitness cost were evaluated. Although two fragments from the *pbp1a^28^* gene were able to confer resistance, only A2 compensated the fitness cost caused by the *pbp2b^28^* mutation. The amino acid sequence deduced from the DNA sequence of A2 revealed that the E285Q substitution might be involved in the compensatory mechanism.

On the other hand, when *pbp2x* alleles from different clinical strains were used to transform *pbp2b^28^*, only one of them (*pbp2x^52^*) was unable to ameliorate the fitness cost of this mutant. Four *pbp2x* alleles shared 9 substitutions compared with Cp1015, with the exception of *pbp2x^52^*, which differed in only 1 residue (D278 in the place of N278). This result suggests that N278, present in the *pbp2x* mutations that compensated the fitness cost of *pbp2b^28^*, could be also a compensatory mutation, at least in the set of mutations described for *pbp2x^12^*, *pbp2x^52^*, *and pbp2x^62^*.

Rieux and coworkers [11] studied the relationship between the acquisition of penicillin resistance and virulence, and demonstrated that the single and double *pbp2b pbp2x* mutants lost virulence in an intraperitoneal infection model in Swiss mice. After several passages in mice, the *pbp2x* mutations showed stability. However, for the *pbp2b* mutants, virulent revertants were recovered without intragenic modifications, suggesting that extragenic compensatory mutations were involved in this phenomenon. On the other hand, Trzcinski and coworkers [12] reported a rise in the βL resistance levels of the susceptible D39 strain, by the sequential transformation of the *pbp* genes obtained from clinical isolates. This event was accompanied by an increase in the fitness cost of a triple *pbp* mutant as determined by a rat nasal colonization model, but without growth alterations occurring in vitro. However, in the Trzcinski's work, no fitness compensation was detected among the *pbp* mutations obtained from isolates belonging to the Spain^6B^-2, Hungary^19A^-6, or serotype-9V Spain^9V^-3 international clones.

In contrast with these reports, we showed that *pbp2x* and *pbp1a* genes carrying specific mutations that conferred βL resistance, not only compensated the *pbp2b*-associated fitness cost in Cp1015 and D39 strains, but also increased the βL resistance levels. We also demonstrated that this that this compensatory mechanism was present in the clinical strains that belonged to the new serotype-14 variant of the Spain^9V^-3 pneumococcal clone identified in Argentina [13]. Related to this, several other studies have reported that the fitness cost associated to antibiotic resistant mutants depends on the specific mutations related to the resistant mechanism and the genetic background [5], [24], [25], [26], [27], [28]. Here, we also used strain D39, the same strain used by Trzcinski and coworkers [12], as an acceptor for *pbp* transformations. However, contrasting results were obtained, which might be explained by considering the different nature of the *pbp* mutations and the particular evolution of the clinical isolates carrying these mutations. These assumptions were supported by an amino acid sequence analysis of the PBPs encoded by the mutated *pbp2b*, *pbp1a* and *pbp2x* genes from the clinical strains used in this study, with and a clear difference being found with those described by Trzcinski and coworkers [12].

When the βL resistance of the double and triple *pbp* mutants was analyzed, we found an increase not only in its level, but also in its spectrum. We postulate a direct correlation between an increase in βL resistance and fitness cost compensation. A similar mechanism was previously shown for fluoroquinolone resistance, by analyzing the *parC/E* and *gyrA* mutations obtained from clinical pneumococcal strains using competitive fitness assays in vitro [8]. Recently, this phenomenon was also described for fluoroquinolone resistance in *E. coli* by fitness assays in vitro and in vivo using a laboratory strain [28].

Although the main focus of the present work was the compensatory evolution associated to the *pbp* mutations that conferred βL resistance, it is important to highlight another topic linked to PBPs, the cell division process. In particular, we studied the putative mechanism that caused morphological changes in *pbp2b* mutants. It is already known that PBPs play an important role in cell wall synthesis [29], but morphological alterations have not been previously described for *pbp* mutants in the absence of β-lactams. In this work, we described an atypical transition from a coccoid to a rod-like shape and also other alterations caused by *pbp2b* mutated genes obtained from βL^R^ clinical isolates, with this morphological phenotype being reproduced in different laboratory strains. In a previous work, it was proposed that PBP2b, an essential class-B PBP, is involved in peripheral peptidoglycan synthesis in *S. pneumoniae* [16]. Related to this, bacterial cell morphogenesis is regulated by a controlled peptidoglycan synthesis, where PBP activity is essential for the normal progression of this process [30], and also by an orchestrated interaction of cytoskeleton proteins triggered by FtsZ polymerization [31]. The septal accumulation observed by Van-FL staining in the *pbp2b* mutants indicated an abnormal cell division. To investigate the impact of *pbp2b* mutations on this process, we analyzed the FtsZ localization by immunofluorescence in the *pbp2b* mutant and found an unusual localization pattern, revealing structures that resembled the helical Van-FL-stained sidewall shown by *Bacillus subtilis* [17]. To search for the reason why the *pbp2b* mutants showed these morphological alterations, we also measured the stability of PBP2b^28^ and found that PBP2b^28^ showed an increased half-life, in contrast with that observed in the wild-type strain. The fact that PBP2b^28^ displayed a higher protein level led us to investigate a putative delocalization of this protein in a *pbp2b^28^* genetic background. Immunofluorescence assays showed that PBP2b-GFP fusion localized equatorially as FtsZ did in the wild-type strain described by Morlot and coworkers [16]. In contrast, PBP2b^28^-GFP fusion revealed an atypical helical distribution similar to FtsZ in the *pbp2b^28^* mutant. It is possible that protein-protein interactions may have altered the localization of certain proteins, and we suspected that PBP2b^28^ could have interacted with FtsZ, thus modifying its normal emplacement. Using a bacterial two-hybrid system, we demonstrated that FtsZ was able to interact with PBP2b^28^ as well as with the wild-type protein, but not with PBP2x. Considering that PBP2b^28^ was delocalized, we propose that this interaction of PBP2b^28^ with FtsZ led to a misplacement of this protein, and consequently, to an aberrant cellular morphology that caused a decreased fitness. Moreover, these results support the idea that this interaction is essential for the control of cell morphogenesis in *S. pneumoniae*, as suggested for *E. coli* [32], [33]. In addition to this phenomenon, we suspect that PBP2b^28^ presented a decreased transpeptidase activity, as all the PBP mutants confer βL^R^ by a decreased affinity to these compounds [34], [35]. Because PBP2b has been involved in the elongation stage during cell division [16], we propose that as PBP2b^28^ could not elongate efficiently then these cells accumulated septa, as demonstrated by Van-FL staining assays, and this could be another cause of the morphological alterations found in the *pbp2b^28^* mutant. In our laboratory, work is now in progress in an attempt to gain a better understanding of this complex process and this not well understood molecular mechanism.

To try to explain the putative cause of compensation of the PBP2b^28^ alterations by PBP1a^28^ and PBP2x^28^, we also analyzed the stability of both mutant proteins. We observed that PBP1a^28^ and PBP2x^28^ displayed an increased half-life in the triple *pbp* mutant as we had also for PBP2b^28^. Considering that all these PBPs have transpeptidase activity, these results suggest that PBP1a^28^ and PBP2x^28^ have a complementary function on PBP2b^28^, based mainly on their increased protein levels. We propose that this event could have caused the compensatory effect on fitness and the morphological alterations in the *pbp2b^28^* mutant.

In the current work, we reported compensatory extragenic mutations that restored the fitness alterations imposed by mutations related to βL resistance. Given that the fitness compensation determined by assays in vivo and in vitro were similar, our data suggest that the selection of fitter strains may have taken place in the natural habitat of *S. pneumoniae*. This phenomenon has an important clinical relevance, since these compensated *pbp* mutants cannot only maintain but also increase the βL resistance levels, in contrast with previous reports [22]. Our results also indicate that clinical strains acquired those *pbp* mutations which improved their resistance-associated fitness cost, producing a clear competitive and selective advantage, and thus raising the potential spreading of βL resistance. We think it is probable that *S. pneumoniae* exploited its high transformability to acquire the compensatory *pbp* mutations that are essential for its persistence and dissemination. This idea is supported by the experimental data, which indicated that the *pbp* mutants showed no transformability alterations, and that the *pbp* mutants could be selected at a transformation rate higher than 10^-2^/µg of DNA, thus demonstrating that compensatory mutations can be acquired in a one-step transformation. If this compensatory evolution was due to the sequential incorporation of adaptive mutations, then the occurrence of this phenomenon may increase rapidly under antibiotic pressure. Therefore, it is possible that certain mutations were selected for their compensatory effect on fitness in addition to their contribution in developing higher βL resistance levels.

It is known that the emergence and stability of antibiotic resistance is a complex biological process, being driven by different factors such as the volume of antibiotic used, the rate of resistant mutant formation, the fitness cost imposed and the compensatory mechanisms to improve that cost [22]. Many studies on the effect of a reduction in β-lactam consumption have reported a sustained resistance level to *S. pneumoniae* [22], suggesting that other factors in addition to antibiotic exposition are contributing to the persistence and the evolution of βL resistance. In the present work, the compensatory mechanism seems to be an important factor, favoring environmental long-term persistence and the spreading of βL^R^ strains. Moreover, it is known that fitness compensation improves the dissemination ability of resistant strains, an essential trait that characterizes pneumococcal clones. However, we could not show that this phenomenon was particularly associated to the *pbp* genes for other successful international clones. As mentioned above, fitness restoration has not been reported for *pbp* mutations obtained from clinical strains that belong to the Spain^6B^-2, Hungary^19A^-6, and serotype-9V Spain^9V^-3 international clones [12]. Nevertheless, we demonstrated that a compensatory mechanism was present, at least in the new serotype-14 variant of the Spain^9V^-3 clone recently characterized in our laboratory [13], and also in a serotype-9V reference strain of this clone (ATCC 700671) isolated in France [36]. Therefore, this model contributes to the understanding of βL resistance evolution in *S. pneumoniae*.

## Materials and Methods

### Bacterial strains, growth conditions and susceptibility testing procedures

The bacterial strains used in this work are listed in Table S1 in Supporting Information S2. βL^R^ pneumococcal strains were obtained from invasive infections of pediatric patients and belonged to the Spain^9V^-3 international clone [13]. Cells were routinely grown at 37°C in Todd-Hewitt broth supplemented with 1% bovine serum albumin. For antimicrobial susceptibility testing, strains were grown at 37°C in a 5% CO_2_ atmosphere on Mueller-Hinton agar with 5% defibrinated sheep blood. Penicillin, cefotaxime, and piperacillin MICs were determined by agar dilution following a CLSI protocol [37].

### PCR conditions and transformation assays

To amplify the transpeptidase region of the *pbp1a*, *pbp2b*, and *pbp2x* genes by PCR, we used the F1a/R1a, F2b/R2b, and F2x/R2x [13] primer pairs, respectively (Fig. S1 in Supporting Information S1). The internal fragments of the *pbp1a* gene were amplified and sequenced with the following primer pairs: Fa1/Ra1 (from position 94 to 234); Fa2/Ra2 (from position 599 to 1002), Fa3/Ra3 (from position 881 to 1294), Fa4/Ra4 (from position 385 to 521) and Fa5-Ra5 (from position 476 to 629) (Fig. S6 in Supporting Information S1). The internal fragments of the *pbp2x* gene were amplified and sequenced with the following pair primers: Fx1/Rx1 (from position 125 to 271), Fx2/Rx2 (from position 238 to 370); Fx3/Rx3 (from position 320 to 469) and Fx4-Rx4 (from position 445 to 738) (Fig. S7 in Supporting Information S1). The internal fragments of the *pbp2b* gene were amplified and sequenced with the following primer pairs: Fb1/Rb1 (from upstream of *pbp2b* to 114); Fb2/Rb2 (from position 69 to 256); Fb3/Rb3 (from position 217 to 439); Fb4/Rb4 (from position 374 to 557) and Fb5/Rb5 (from position 502 to downstream *pbp2b*) (Fig. S17 in Supporting Information S1). The primers sequences are detailed in Table S4 in Supporting Information S2. PCR products were amplified using the following parameters: initial denaturation at 94°C for 4 min, 30 cycles of denaturation at 94°C for 45 s, annealing at 55°C for 30 s, elongation at 72°C for 1 min, and a final extension at 72°C for 10 min. Chromosomal DNA was isolated using the Wizard Genomic DNA purification kit (Promega), following the manufacturer's instructions. The PCR products were amplified from chromosomal DNA and used to genetically transform the Cp1015 and D39 strains as described previously [38]. Transformants were selected on Mueller-Hinton agar plates supplemented with 5% defibrinated sheep blood containing 0.05 µg/ml piperacillin or 0.1–0.3 µg/ml cefotaxime. Natural competence was performed as described previously (3).

### Flow cytometry analysis

Strain Cp1015 and its respective *pbp* mutants were individually grown to the mid-logarithmic phase in Todd-Hewitt broth at 37°C. Cells were then washed three times and resuspended in PBS. In order to analyze cell shape modifications by flow cytometry analysis, bacteria were injected into a FACS Aria. Between 50,000 and 100,000 events were counted and analyzed by using WinMDI software. To compare populations, the data for each strain were plotted on a two-dimensional graph (*x-*axis, forward scatter; *y-*axis, side scatter).

### Determination of relative fitness in vitro

Relative fitness was quantified as the average number of surviving progeny of a particular genotype, and this was compared with average number of surviving progeny of competing genotypes after a single generation. The wild-type strain genotype was normalized at wt = 1 and the fitnesses of other genotypes were measured with respect to that genotype [39]. The cost of a resistance mutation was determined by direct competition against the susceptible Cp1015 strain as described previously [7]. Individual strains were exponentially grown to an OD_600 nm_ of 0.2 and cultures were diluted 2,000-fold. Mixed cultures containing equivalent amounts of the Cp1015 and *pbp* mutant cells (about 5×10^4^ CFU/ml) were incubated in antibiotic-free medium for 6 h. These mixed cultures were then diluted 1,000-fold to avoid the typical lysis of *S. pneumoniae* cultures at the stationary phase, and cells were cultured for an additional 6 h. The number of viable cells was determined at 0, 6 and 12 h by plating serial dilutions of the culture on BHI agar with 5% defibrinated sheep blood, containing 0.05 µg/ml piperacillin (for *pbp2b* simple mutants), 0.1 µg/ml cefotaxime (for *pbp* double and triple mutants), or with no antibiotic. The number of susceptible cells was calculated by subtracting the number of resistant cells from the total cell number revealed by the CFU counts of the plates without drug. To determine the CFU numbers, the mean of four counts was calculated. The number of generations of the resistant and Cp1015 strains in the mixed culture was calculated by using the following formula: (log B - log A)/(log 2), where A is the number of CFU/ml at time zero and B is the number of CFU/ml at the end of each cycle (6 h and 12 h). The relative fitness of each strain was determined from the ratio of the number of generations of the resistant strain and Cp1015. The mean of four to nine replicate competition assays were determined. Statistical tests were performed using Instat software.

### Determination of relative fitness in vivo

These assays using a mice model were performed as described previously [40], but with specific modifications for *S. pneumoniae*. Seven male C57BL/6, 4 to 5 weeks old (obtained from Comision Nacional de Energia Atomica, Ezeiza, Argentina), were inoculated intraperitoneally (under isoflurane anesthesia) with a mix of equal parts of D39 strain and an isogenic *pbp* mutant (1×10^5^ CFU) in 0.1 ml 50 mM glucose (in PBS). These mice were sacrificed by CO_2_ asphyxiation after 2 days, and strains were recovered from each homogenized liver by plating onto BHI agar containing piperacillin, cefotaxime, or no antibiotic, as described above. Relative fitness was determined from the ratio of the number of generations of the resistant strains and strain D39. ANOVA statistical tests were performed with Instat software.

### Ethics statement

This study was carried out in strict accordance with the recommendations in the Guide to the Care and Use of Experimental Animals published by the Canadian Council on Animal Care. The experimental protocols performed were reviewed and approved by the Ethic Committee of the Facultad de Ciencias Químicas, Universidad Nacional de Cordoba (Permit number 15-07-68964). The Ethic Committee is constituted by the following professors of the Facultad de Ciencias Químicas-UNC: Laura Chiapello, Claudia Sotomayor, Margarita Briñon, Teresa Scimonelli, Santiago Quiroga, Mariana Contin and Graciela Granero. The protocols and the Animal Laboratory of our Institute (CIBICI-CONICET, Argentina) obtained an Animal Welfare Assurance from NIH, USA (Assurance number A5802-01, see http://grants.nih.gov/grants/olaw/assurance/500index.htm?Country=AR#GridTop).

All the clinical strains used in this work were provided by the strain collection of the Centro de Estudios Avanzados en Pediatría (Córdoba, Argentina). The Ethics Committee declared in writing that no formal ethical approval was needed to use these clinically obtained materials, because the specimens were remnant from patient samples of blood and nasopharyngeal swabs collected from routine analysis in bacteriology laboratories of public hospitals, and the data were analyzed anonymously.

### Van-Fl staining

Pneumococcal strains were routinely grown to an OD_600 nm_ of 0.2 in Todd-Hewitt broth. Samples were collected and incubated with 2 µg/ml Van-FL (Molecular Probes) for 20 minutes at 37°C. Cells were centrifuged, washed three times with PBS and fixed with 3% paraformaldehyde. After several washes with PBS, cells were spotted on glass slides, air dried, dipped in methanol at −20°C for 10 min, and allowed to dry at room temperature. The cells were then stained with DAPI (Sigma) at a final concentration of 0.2 µg/ml for 10 min before being observed, and images were acquired using a Nikon Eclipse TE-2000 epifluorescence microscope fitted with a Nikon Digital Sight DS-U1 camera. This was performed using ACT-U software, and images were processed with Adobe Photoshop CS version.

### Electron microscopy

Cp1015 and the isogenic mutants *pbp2b^28^*, *pbp2b^28^pbp2x^28^*, and *pbp2b^28^pbp2x^28^pbp1a* were exponentially grown at 37°C in Todd-Hewitt medium, and samples for electron microscopy were then collected, centrifuged, and fixed with 4% formaldehyde-2% formalin in 0.1 M cacodylate buffer for 1 hour at room temperature. An additional fixation with 1% osmium tetroxide in cacodylate buffer was carried out for 1 hour at room temperature. These fixed cells were dehydrated using an increasing concentration of acetone, and embedded in polymerized Araldite at 60°C for 48 hours. Thin sections were obtained using a JEOL JUM-7 microtome equipped with a glass or gem grade diamond knife, and microphotography was performed with a Zeiss LEO 906E microscope.

### Immunolocalization of FtsZ protein

Exponentially growing cells (OD_600 nm_ of 0.25) were fixed with 3% paraformaldehyde for 15 min at room temperature and incubated for 45 min on ice. The fixed bacteria were washed three times in PBS (pH 7.4) and resuspended in GTE (50 mM glucose, 20 mM Tris-HCl, 10 mM EDTA), with a freshly prepared lysozyme solution in GTE being added to a final concentration of 2 µg/ml. 10 µl samples were immediately distributed onto poly-L-lysine microscope slides and air dried. The slides were dipped in −20°C methanol for 5 min, in −20°C acetone for 30 s, and then allowed to dry completely. After rehydration with PBS, the slides were blocked for 1 hour at 37°C with 2% bovine serum albumin (Sigma) in PBS (BSA-PBS). Cells were then incubated with a 1∶100 dilution in BSA-PBS of rabbit polyclonal anti-FtsZ antibody [41] for 1 hour at 37°C. After washing 10 times with PBS, samples were incubated with a fluorescein-conjugated secondary antibody in PBS (Alexa Fluor 488, Molecular Probes) for 30 min at 37°C in the dark. After removing the secondary antibody by washing samples several times with PBS, DAPI was added at a final concentration of 0.2 µg/ml and the samples were incubated for 10 min at room temperature. Slides were washed again, allowed to dry, and then mounted using Dako fluorescent mounting medium (Invitrogen). The final slides were stored at −20°C for several days. Images were acquired using a Nikon Eclipse TE 2000 epifluorescence microscope, and processed with Adobe Photoshop CS version.

### Construction of strains expressing GFP-PBP2b

The *pbp2b* gene was amplified from Cp1015 *wt* and Cba-28 clinical strains with primers F2bf and R2bf (Table S4 in Supporting Information S2). The *gfpmut3* gene was amplified from the pCM18 [42] plasmid using the primer pair Fgfp3/Rgfp3 (Table S4 in Supporting Information S2) in order to obtain a *gfp* copy under the control of the pneumococcal constitutive promoter, Pc [43]. The *pbp* genes were cloned into pGEMT-easy (Promega) and the *gfpmut3* gene into pCRTOPO 2.1 (Invitrogen), thereby generating the pGEM-*pbp* and pGFPTOPO plasmids, respectively (Table S4 in Supporting Information S2). The *pbp* genes were exscinded by *Xho*I digestion of pGEM-*pbp* and inserted into the *Sal*I-restricted pGFP-TOPO plasmid. The *Sal*I site was located in pGFP-TOPO upstream the transcription origin of *gfpmut3* and downstream the Pc. The resultant plasmid pGFP-TOPO-*pbp* contained a *pbp2b* N-terminal fusion to the *gfpmut3* gene under the control of Pc. This construction was then removed from pGFP-TOPO-*pbp* by *EcoR*I digestion and inserted into the same restriction site in the *E. coli-S. pneumoniae* shuttle vector plasmid pAT18 [44]. The resultant vector was named pAT18*pbp-gfp*, and was used to transform the wild-type strain (Cp1015) and the Cp1015 *pbp2b* mutants. The transformant selection was made in BHI agar plates supplemented with 5% blood sheep and 2.5 µg/ml erythromycin. All constructions were confirmed by PCR and sequencing with the same primers used for the amplification of each gene.

### Construction of strains expressing HA-tagged PBPs

Insertion duplication mutagenesis was used to construct strains expressing HA-tagged PBPs. Primer pairs F2btag/R2bHA, F2xtag/R2xHA and F1atag/R1aHA (Table S4 in Supporting Information S2) were designed to amplify approximately the last 300–400 bp of the *pbp2b*, *pbp2x* and *pbp1a* genes fused to the sequence of the HA tag. The PCR products were cloned into the *Xho*I and *Xba*I sites of plasmid pEVP3 [43], and the final constructions (named pEVP32bHA, pEVP32xHA, pEVP31aHA) were used to transform the strains Cp1015, Cp1015 *pbp2b^28^* and Cp1015 *pbp2b^28^ pbp2x^28^ pbp1a^28^*. Selection of the transformants was made in BHI 5% sheep blood agar supplemented with chloramphenicol (2 µg/ml). All constructions were confirmed by PCR and sequencing with the external primers for amplified regions, as mentioned above.

### Protein stability analysis of PBPs

Protein expression was inhibited in exponentially growing cultures in Todd Hewitt broth with 0.5% Yeast Extract (total volume 400 ml) at an OD_600nm_ of 0.2 by the addition of kanamycin (500 µg/ml). One-hundred ml aliquots were withdrawn at 0, 30, 60 and 120 min after the addition of the antibiotic. Cells were immediately harvested, washed once with 1 ml of ice cold PBS and resuspended in 400 µl of ice cold MQ water plus Complete protease inhibitor cocktail (Roche), before being subjected to five freeze-thaw cycles followed by sonication using a Sonics VibraCell VCX130 sonicator (20 cycles, 30 s ON 30 s OFF, 40% amplitude). To solubilize the membrane proteins, 100 µl of 5X RIPA buffer (250 mM Tris pH 7.4, 750 mM NaCl, 5%NP-40, 2.5% Sodium deoxycholate, 0.5% SDS) were added and the samples were heated for 10 min at 95°C, and cell debris was removed by centrifugation for 15 min at 20,000 *g*. The total protein content was determined by using bicinchoninic acid assay [45]. After sodium dodecyl sulfate-polyacrylamide gel electrophoresis and transfer to nitrocellulose membranes of 25 µg of the protein extract, the membranes were probed with a mouse anti-HA primary antibody (1∶2,000; Abacam) and with a goat anti-mouse immunoglobulin G secondary antibody conjugated to horseradish peroxidase (1∶2,500; Invitrogen). Detection was performed with an enhanced chemiluminescence substrate (SuperSignal West Pico Chemiluminescent Substrate; Pierce) and Hyperfilm CL film (GE) using exposures of between 1 and 10 min. Western Blot bands were quantified using the Gel-Pro Analyzer v3.1 software and the half-life was calculated with Graph Pad Prism v5.3 software.

### Protein-protein interaction assays

The bacterial two-hybrid system BacterioMatch II (Stratagene) was used to screen for interactions of PBP2b with FtsZ protein and PBP2x. The *pbp2b* coding sequence was amplified by PCR with the primer pair F2bdh/R2bdh (Table S4 in Supporting Information S2) from the Cp1015 and Cba-28 strains, digested with *Xho*I/*Bam*HI and cloned into the *Xho*I/*Bam*HI restricted bacterial two-hybrid vector pBT. In addition, the coding sequences of *ftsZ* and *pbp2x* were amplified by PCR with F2xdh/R2xdh, FftsZdh/RftsZdh (Table S4 in Supporting Information S2), respectively, digested with *Xho*I/*Bam*HI or *Xho*I/*Bgl*II, in that order, and cloned into the pTRG vector cleaved by *Xho*I/*Bam*HI. The *E. coli* XL1 Blue MR, the BacterioMatch II Validation Reporter strain (Stratagene), was co-transformed with the two plasmids. Growth cultures of the clones on M9 minimal media agar plates containing 2.5 mM 3-amino-1,2,4-triazol (3-AT) and 10 µg/ml of streptomycin were assessed according to the manufacturer's instructions.

## Supporting Information

Supporting Information S1 Supplemental Figures S1 through S17. **Figure S1** Graphical representation of the PBP genes. **Figure S2** Effect of *pbp* mutations on growth curves. **Figure S3** Natural competence assay of *pbp* mutants. **Figure S4** Amino acid substitutions found in PBP2b proteins obtained from different clinical strains. **Figure S5** Amino acid substitutions found in PBP2x proteins obtained from different clinical strains. **Figure S6** Graphical representation of the *pbp1a* gene. **Figure S7** Graphical representation of the *pbp2x* gene. **Figure S8** Amino acid substitutions found in the transpeptidase region of PBP1a^28^ in the *pbp2b^28^ pbp1a^28^* mutant. **Figure S9** Comparison of amino acid sequences of PBP2b mutants. **Figure S10** Comparison of amino acid sequences of PBP2x mutants. **Figure S11** Comparison of amino acid sequences of PBP1a mutants. **Figure S12** Electron microscopy analysis of cellular morphology of strain Cp1015 (*wt)*. **Figure S13** Electron microscopy analysis of the effect of *pbp2b* mutations on cellular morphology. **Figure S14** Electron microscopy analysis of the effect of *pbp2b pbp2X* mutations on cellular morphology. **Figure S15** Electron microscopy analysis of the effect of *pbp2b pbp2x pbp1a* mutations on cellular morphology. **Figure S16** Peptidoglycan synthesis of strain Cp1015 (*wt*) at different stages in the cell cycle. **Figure S17** Graphical representation of the *pbp2b* gene showing the different DNA fragments sequenced.
(PDF)

Supporting Information S2 Supplemental Tables S1 through S4. **Table S1** Bacterial strains and plasmids used in this work. **Table S2** Fitness cost analysis of double *pbp2b pbp2x* mutants obtained with *pbp2x* genes from different clinical strains. **Table S3** Contribution of internal fragments of *pbp1a* and *pbp2x* in resistance and fitness compensation. **Table S4** Primers used in this work.
(PDF)
