# Supplementary material for: Compensatory Evolution of pbp Mutations Restores the Fitness Cost Imposed by β-Lactam Resistance in Streptococcus pneumoniae
Source: PLoS Pathog. 2011 Feb 17;7(2):e1002000. doi: 10.1371/journal.ppat.1002000 (PMC3040684; doi:10.1371/journal.ppat.1002000)
Supplement: Supporting Information S1 — Supplemental Figures S1 through S17. Figure S1 Graphical representation of the PBP genes. Figure S2 Effect of pbp mutations on growth curves. Figure S3 Natural competence assay of pbp mutants. Figure S4 Amino acid substitutions found in PBP2b proteins obtained from different clinical strains. Figure S5 Amino acid substitutions found in PBP2x proteins obtained from different clinical strains. Figure S6 Graphical representation of the pbp1a gene. Figure S7 Graphical representation of the pbp2x gene. Figure S8 Amino acid substitutions found in the transpeptidase region of PBP1a28 in the pbp2b28 pbp1a28 mutant. Figure S9 Comparison of amino acid sequences of PBP2b mutants. Figure S10 Comparison of amino acid sequences of PBP2x mutants. Figure S11 Comparison of amino acid sequences of PBP1a mutants. Figure S12 Electron microscopy analysis of cellular morphology of strain Cp1015 (wt). Figure S13 Electron microscopy analysis of the effect of pbp2b mutations on cellular morphology. Figure S14 Electron microscopy analysis of the effect of pbp2b pbp2X mutations on cellular morphology. Figure S15 Electron microscopy analysis of the effect of pbp2b pbp2x pbp1a mutations on cellular morphology. Figure S16 Peptidoglycan synthesis of strain Cp1015 (wt) at different stages in the cell cycle. Figure S17 Graphical representation of the pbp2b gene showing the different DNA fragments sequenced. (PDF) [file ppat.1002000.s001.pdf]

Supporting Information

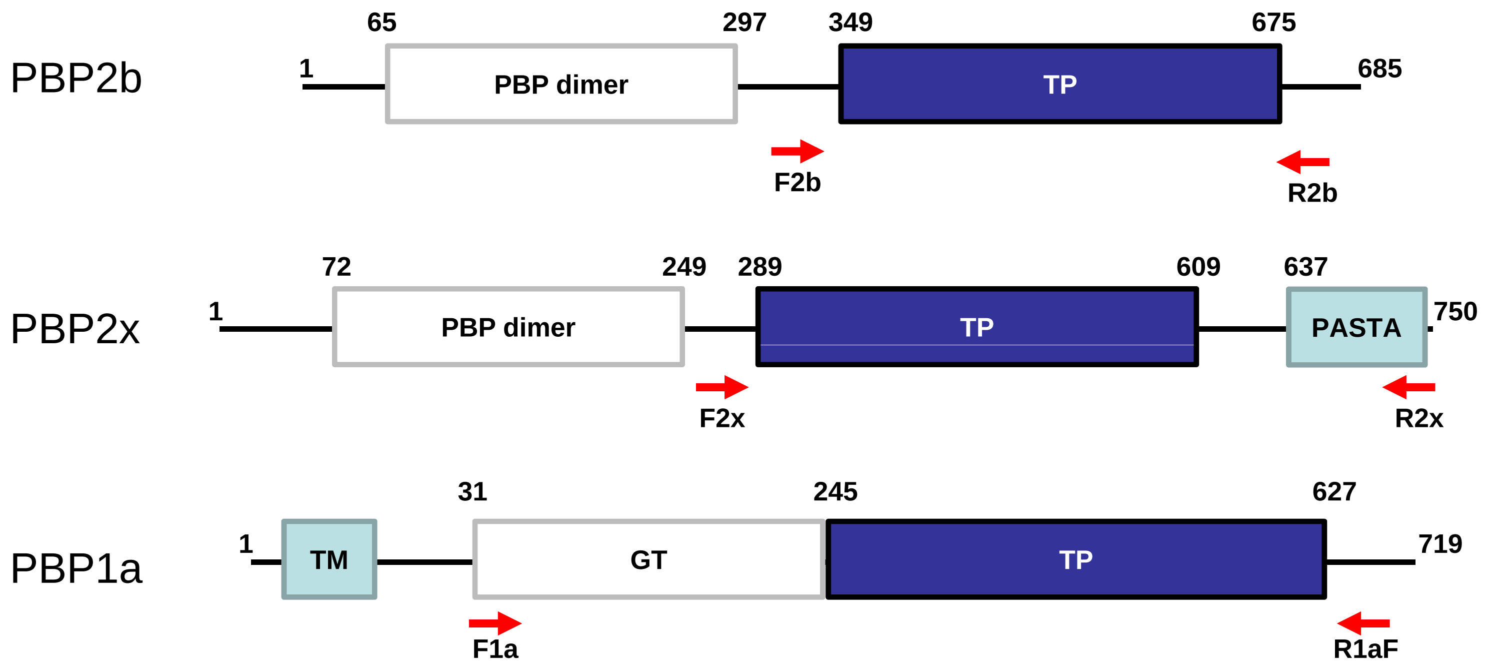

Fig. S1. Graphical representation of the *pbp* genes showing the different amplified regions used to transform the Cp1015 strain to obtain single, double and triple *pbp* mutants. The arrows represent the position of the primers used to amplify each *pbp* region.

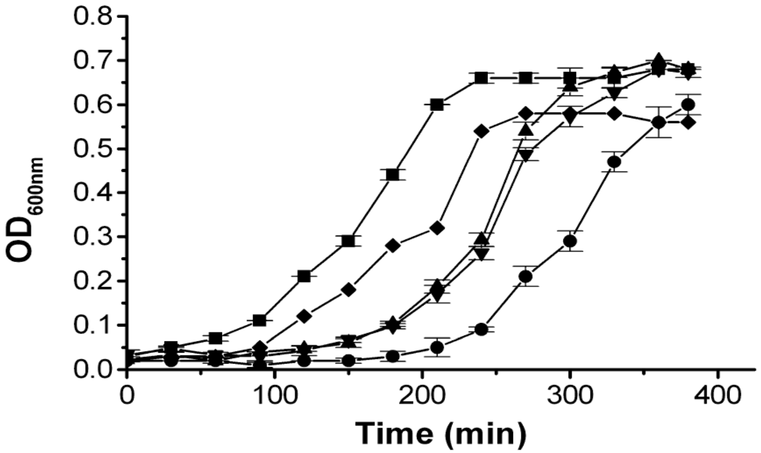

Fig. S2. Effect of *pbp* mutations on growth curves. Growth curves of *S. pneumoniae* were incubated in Todd Hewitt broth supplemented with 0.01% albumin at 37ºC for CP1015 *wt* (■), CP1015 *pbp2b*[28] (●), CP1015 *pbp2b*[28] *pbp1a*[28] (▼), CP1015 *pbp2b*[28] *pbp2x*[28] (▲), and CP1015 *pbp2b*[28] *pbp2x*[28] *pbp1a*[28] (♦).

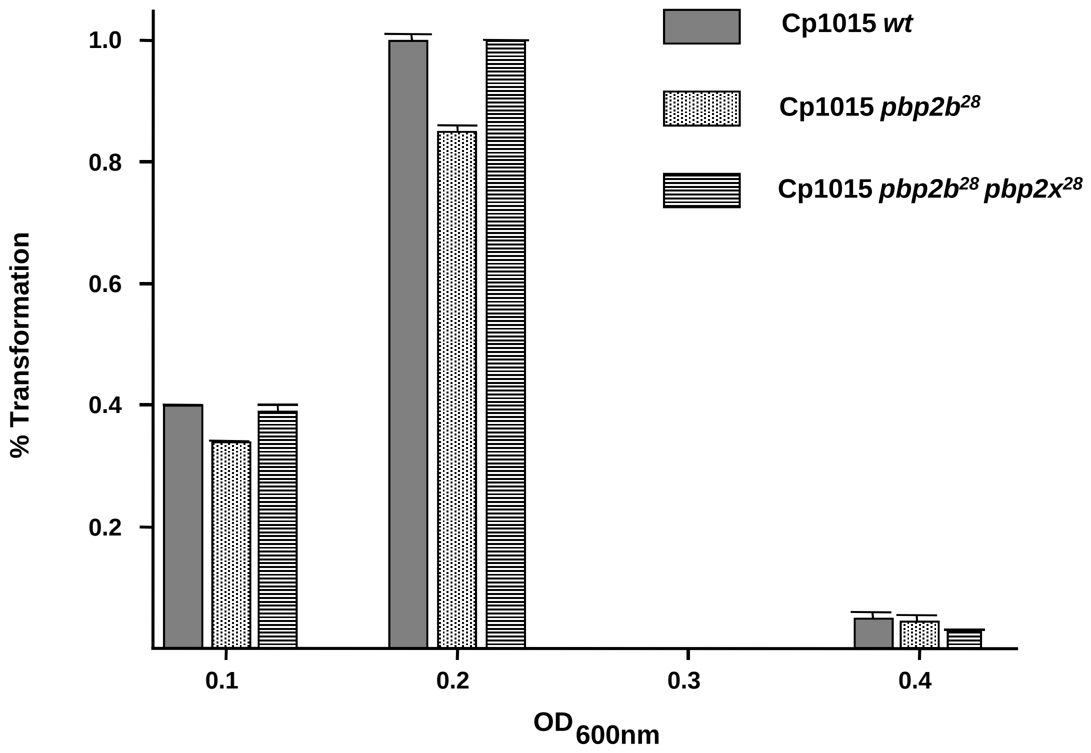

Fig. S3. Natural competence assay. *S pneumoniae* Cp1015 *wt*, *pbp2b*, and *pbp2b pbp2x* mutants were grown in CTM medium. The percentage of transformation (ratio of cfu on rifampicin agar plates and cfu on agar plates without antibiotic) indicates the bacterial capacity of DNA incorporation and recombination. The *pbp* mutants showed the typical maximum transformation level at OD_{600nm} 0.1-0.2 as found for the Cp1015 *wt* strain.

```
            333333333333333333333333333333444444444444444444444444444444
            777777778888888888999999999900000000001111111111222222222233
            234567890123456789012345678901234567890123456789012345678901
TP2b WT     TGELTPDSLGTVTNVFVPGSVVKAATISSGWENGVLSGNQTLTDQSIVFQGSAPINSWYT
TP2b Sp     .............................................P.........Y...K
TP2b 12     ..Q.SS.....I.................................P..............
TP2b 19     .............................................P.........Y...K
TP2b 28     .............................................P.........Y...K
TP2b 52     .............................................P.........Y...K
TP2b 56     .............................................P..............
TP2b 62     .............................................P.........Y...K

            444444444444444444444444444444444444444444444444444444444444
            333333334444444444555555555566666666667777777777888888888899
            234567890123456789012345678901234567890123456789012345678901
TP2b WT     QAYGSFPITAVQALEYSSNTYMVQTALGLMGQTYQPNMFVGTSNLESAMEKLRSTFGEYG
TP2b Sp     L..........E.......A........I.................T..G...A......
TP2b 12     LS.........E................I.....T...VAA.GQ..T..G..........
TP2b 19     L..........E.......A........I.................T..G...A......
TP2b 28     L..........E.......A........I.................T..G...A......
TP2b 52     L..........E.......A........I.................T..G...A......
TP2b 56     ...........E.......A........I...........L.N......G......A...
TP2b 62     L..........E.......A........I.................T..G...A......

            444444445555555555555555555555555555555555555555555555555555
            999999990000000000111111111122222222223333333333444444444455
            234567890123456789012345678901234567890123456789012345678901
TP2b WT     LGTATGIDLPDESTGFVPKEYSFANYITNAFGQFDNYTPMQLAQYVATIANNGVRVAPRI
TP2b Sp     ..A..........................S..............................
TP2b 12     ..AS............T...FDLG...N.S.........................L..H.
TP2b 19     ..A..........................S..............................
TP2b 28     ..A..........................S..............................
TP2b 52     ..A..........................S..............................
TP2b 56     ..AS............I....N........................G........I..H.
TP2b 62     ..A..........................S..............................

            555555555555555555555555555555555555555555555555
            555555556666666666777777777788888888889999999999
            234567890123456789012345678901234567890123456789
TP2b WT     VEGIYGNNDKGGLGDLIQQLQPTEMNKVNISDSDMSILHQGFYQVAHG
TP2b Sp     ................................................
TP2b 12     .........Q....S.V.ETATK.L......E...A..Q......S..
TP2b 19     ................................................
TP2b 28     ................................................
TP2b 52     ................................................
TP2b 56     ........EQ....N...SVESK....I...E..V...Q......S..
TP2b 62     .....................................................
```

Fig. S4. Amino acid substitutions found in PBP2b proteins obtained from different clinical strains. The sequences shown correspond to the TP region from Cp1015 (WT), Spain^{9V}-3 clone (Sp) and the clinical strains 12, 19, 28, 52, 56 and 62. The Cp1015 sequence is shown as a reference; identical sites are represented by dots, with only polymorphisms being indicated. The amino acid position are indicated vertically. Amino acids in bold represent common substitutions.

```
          2222222222222222222222222222222222222223333333333333333333333333333
          6666666666777777777788888888888999999999990000000000111111111122222222
          1234567890123456789012345678901234567890123456789012345678901234567
TP2X WT   VYTTISSPLQSFMETQMDAFQEKVKGKYMTATLVSAKTGEILATTQRPTFDADTKEGITEDFVWRDI
TP2x Sp   ....L...............L.............................N................
TP2x 12   .......T.........N.......................................L.K.......
TP2x 52   .......T.................................................L.K.......
TP2x 54   .......T.........N.......................................L.K.......
TP2x 56   .........................................................L.K.......
TP2x 62   .......T.........N.......................................L.K.......
TP2x 19   ESN.................L.............................N................
TP2x 28   ESN.................L.............................N................

          3333333333333333333333333333333333333333333333333333333333333333333
          2233333333334444444444555555555566666666667777777777888888888899999
          8901234567890123456789012345678901234567890123456789012345678901234
TP2X WT   LYQSNYEPGSTMKVMMLAAAIDNNTFPGGEVFNSSELKIADATIRDWDVNEGLTGGRMMTFSQGFAH
TP2x Sp   ..........A....T..SS.......S..Y.....F......T......D...T.G....L.....
TP2x 12   ...............T.........................V........D...T......L....L
TP2x 52   ...............T.........................V........D...T......L....L
TP2x 54   ...............T.........................V........D...T......L....L
TP2x 56   ..........A....T......................V..V..............G..........
TP2x 62   ...............T.........................V........D...T......L....L
TP2x 19   ..........A....T..SS.......S..Y.....F......T..........T.G..........
TP2x 28   ..........A....T..SS.......S..Y.....F......T..........T.G..........

          33334444444444444444444444444444444444444444444444444444444444444444
          99990000000000111111111122222222223333333333444444444455555555556666
          56789012345678901234567890123456789012345678901234567890123456789012
TP2X WT   SSNVGMTLLEQKMGDATWLDYLNRFKFGVPTRFGLTDEYAGQLPADNIVNIAQSSFGQGISVTQTQMI
TP2x Sp   ......S...............K..........................S.................L
TP2x 12   ...................................................................L
TP2x 52   ...................................................................L
TP2x 54   ...................................................................L
TP2x 56   ....................................................................
TP2x 62   ...................................................................L
TP2x 19   ......S...............K..........................S.................L
TP2x 28   ......S...............K..........................S.................L
```

Fig. S5. Amino acid substitutions found in PBP2x proteins obtained from different clinical strains. The sequences shown were obtained from Cp1015 (WT), Spain^{9V}-3 clone (Sp) and the clinical strains Cba-12, Cba-52, Cba-56 and Cba-62. Identical sites in other strains are represented by dots, with only polymorphisms being indicated. The amino acid position of the polymorphic sites is indicated vertically. The N278 substitution is shown in bold.

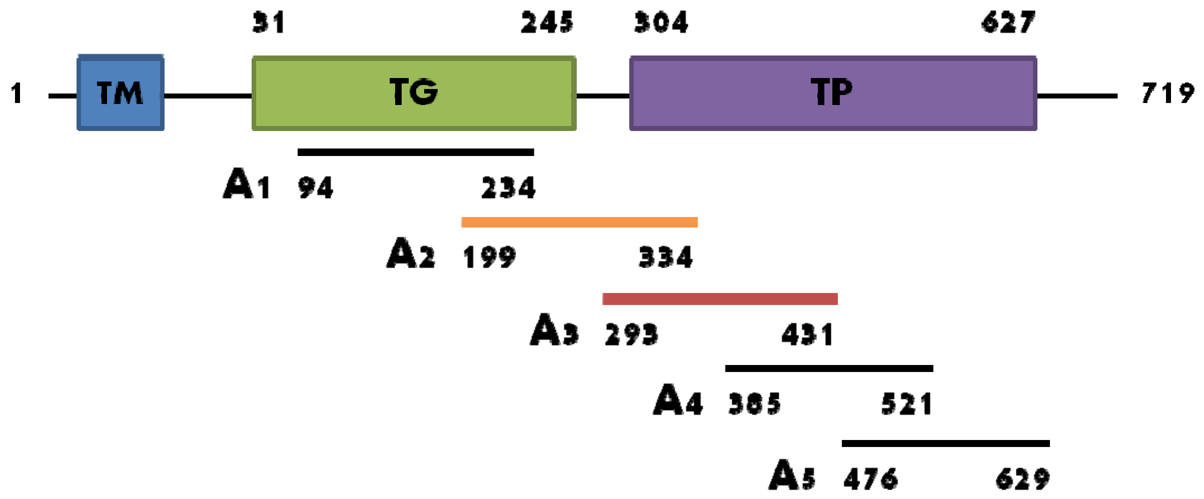

Fig. S6. Graphical representation of the *pbp1a* gene showing the different regions used to transform the Cp1015 *pbp2b*[28] mutant to obtain the Cp1015 *pbp2b*[28] *pbp1a*[28]-A2 and Cp1015 *pbp2b*[28] *pbp1a*[28]-A3 mutants. A2 and A3 sequence analysis allowed the evaluation of the contribution of amino acidic substitutions in cefotaxime resistance and *fitness* compensation.

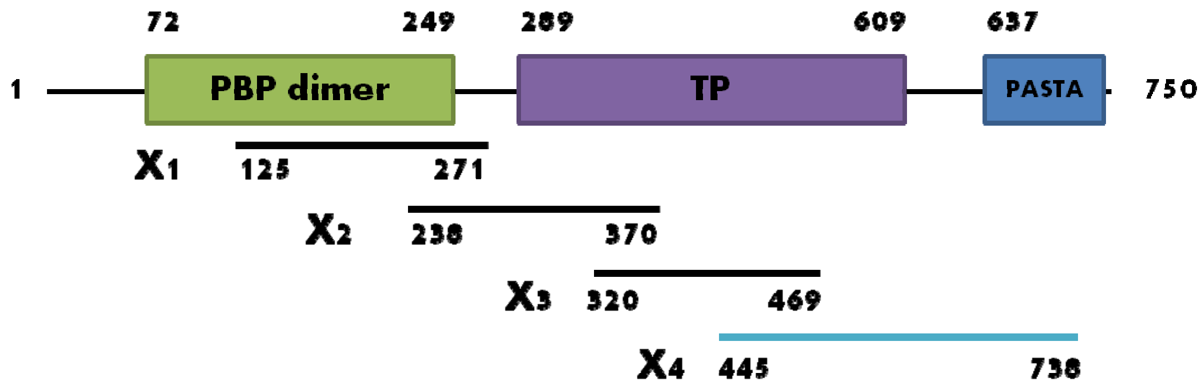

Fig. S7. Graphical representation of the *pbp2x* gene showing the region used to transform the Cp1015 *pbp2b*^{28} mutant to obtain the Cp1015 *pbp2b*^{28} *pbp2x*^{28} -X4mutant. The X4 sequence analysis allowed the evaluation of the contribution of amino acidic substitutions in cefotaxime resistance and *fitness* compensation.

```
                          A2                                   A3
        2222222222222222222333333333333333333333333333333333333333333333333
        8888888889999999999000000000011111111112222222333333344444444445555
        1234567890123456789012345678901234567890123456345678901234567890123
R6      NQVEEETGYNLLTTGMDVYTNVDQEAQKHLWDIYNTDEYVAYPDDEIVDVSNGKVIAQLGARHQSSN
WT      ...................................................................
28      ....Q..............................S.Q..S....DV.................A..
WT-A*   ....Q..............................S.Q..S....DV.................A..

                                     A3
        3333333333333333333333333333333333333333333333444444444444444444444
        5555556666666666777777777788888888889999999999000000000011111111112
        4567890123456789012345678901234567890123456789012345678901234567890
R6      VSFGINQAVETNRDWGSTMKPITDYAPALEYGVYESTATIVHDEPYNYPGTNTPVYNWDRGYFGNIT
WT      ..................................D................................
28      ....T............A..........I.....D....M.N.I.......S........A......
WT-A*   ....T............A..........I.....D....M.N.I.......S........A......
```

Fig. S8. Amino acid substitutions found in the transpeptidase region of PBP1a in the *pbp2b*[28] *pbp1a*[28] mutant. The amino acid sequences shown correspond to the A2 and A3 regions from Cp1015 (WT), strain Cba-28 (28) and the Cp1015 *pbp2b*[28] A2/A3*pbp1a*[28] mutant (WT-A*). The R6 sequence is shown as a reference; identical sites in other strains are represented by dots, with only the polymorphisms being indicated. The amino acid position of the polymorphic sites is indicated vertically. Amino acids in bold are the substitutions involved in resistance, and the underlined residue is probably related to fitness compensation.

```
             3333444444444444444444444444445555555555556
             9999122233344444445567788888892467777778891
             1234778901734567890108901234541360123463772
             ****               ***
WT           SVVKSNSWYTQQALEYSSNTLSAMEKLRSTANDQLQPTMDSAG
WT-2b19      ....PY...KLE.......AIT......A.S............
WT-2b28      ....PY...KLE.......AIT......A.S............
Cba-19       ....PY...KLE.......AIT..G...AAS............
Cba-28       ....PY...KLE.......AIT..G...AAS............
607T         ....PY...KLE.......AIT..G...AA.DEAIDTKIEAS.
608          ....PY...KLE.......AIT..G...AA.DEAIDTKIEAS.
901T         ....PY...KLE.......AIT..G...AA.............
902          ....PY...KLE.......AIT..G...AAS............
```

Fig. S9. Comparison of amino acid sequences of PBP2b mutants. The sequences shown correspond to the TP region from the Cp1015 (WT), Cba-19 and Cba-28 clinical strains, and the Cp1015 *pbp2b*^{19} (WT-2b^{28}) and Cp1015 *pbp2b*^{28} (WT-2b^{28}) mutants. Identical sites in other strains are represented by dots, with only the polymorphisms being indicated. The amino acid position of the polymorphic sites are indicated vertically. Asterisks show the TP active sites. Amino acids in bold and underlined indicate the most common substitutions found in resistant isolates. Sequences are also compared to those described by Trzcinski *et al* (607T, 608, 901T and 908).

```
              222222222333333333333333333333333333333
              666666668113333344444444455555666666666
              123456781125678901234567856789012345678
                           ****
WT            VYTTISSPQDAPGSTMKVMMLAAAIGGEVFNSSELKIAD
WT-2x19       ESS.....LN....A....T..SS.S..Y.....F....
WT-2x28       ESS.....LN....A....T..SS.S..Y.....F....
Cba-19        ESS.....LN....A....T..SS.S..Y.....F....
Cba-28        ESS.....LN....A....T..SS.S..Y.....F....
607T          .......TLN....A....T..SSVS..Y.....F....
608           .......TLN....A....T..SS.S..Y.....F....
901T          ....L...LN....A....T..SS.S..Y.....F....
902           ....L...LN....A....T..SS.S..Y.....F....

              33333333333333333333333334444444444444
              67777888888888899999999990014688889999
              90189012345678901234567890174267890123
                                  ***
WT            ATIEGLTGGRMMTFSQGFAHSSNVGMTNNIPNDQTARK
WT-2x19       ..TD...T.G....L...........SKL.........
WT-2x28       ..TD...T.G....L...........SKL.........
Cba-19        ..TD...T.G....L...........SKL.........
Cba-28        ..TD...T.G....L...........SKL.........
607T          ..TD...T.G....L...........SKSLT.N.SV..
608           ..TD...T.G....L...........SKSLT.N.SV..
901T          ..TD...T.G....L...........SKSLT.N.SV..
902           ..TD...T.G....L...........SKSLT.N.SV..
```

Fig. S10. Comparison of amino acid sequences of PBP2x mutants. The sequences shown correspond to the TP region from the Cp1015 (WT) Cba-19 and Cba-28 clinical strains, and the Cp1015 *pbp2b*^{19} (WT-2x^{19}), and Cp1015 *pbp2b*^{28} (WT-2x^{28}) mutants. Identical sites in other strains are represented by dots, with only the polymorphisms being indicated. The amino acid position of the polymorphic sites are indicated vertically. Asterisks show the TP active sites. Amino acids in bold and underlined indicate the most common substitutions found in resistant isolates. Sequences are also compared to those described by Trzcinski et al (607T, 608, 901T and 908).

```
             3333333333333333333333333333333334444444444444444444444
             2223555555557777777777888899999990122223334555666777999
             5673123456780123456789012812345675467890123789012345567
                               ****                        ***
WT           DELISSNVSFGISTMKPITDYAPALEATIVHDENGQQSRNVPNPSIHYSDKKTYY
WT-1a^19     .D.VA......T.A..........ID..M.N.ISA......TD..M..AN.QI.H
WT-1a^28     .D.VA......T.A..........ID..M.N.ISA......TD..M..AN.QI.H
Cba-19       .D.VA......T.A..........ID..M.N.ISA......TD..M..AN.QI.H
Cba-28       .D.VA......T.A..........ID..M.N.ISA......TD..M..AN.QI.H
607T         .D.VA......T.A..........I...M.N.ISA......TD..M..AN.QI.H
608          .D.VA......T.A..........I...M.N.ISA......TD..M..AN.QI.H
901T         .D.VA......T.A..........I...M.N.ISA......TD..M..AN.Q...
902          .D.VA......T.A..........I...M.N.ISA......TD..M..AN.Q...

              555555555555555555555555555555555555555555555566666666
              011111134455555555556666666666777777777788888800001112
              323456730601234567890123456789012345678901234567890122
                                                ***
WT            HEKEFSNDSNAWLPQAGKTGTSNYTDEEIENHIKTSQFVAPDELFALVGNGLTM
WT-1a^19      NK....DEAGP..A............D...K...NTGY.....M.VI..D.FLI
WT-1a^28      NK....DEAGP..A............D...K...NTGY.....M.VI..D.FLI
Cba-19        NK....DEAGP..A............D...K...NTGY.....M.VI..D.FLI
Cba-28        NK....DEAGP..A............D...K...NTGY.....M.VI..D.FLI
607T          NK....DEAGP..A............D...K...NTGY........I..D.FLI
608           NK....DEAGP..A............D...K...NTGY.....M.VI..D.ELI
901T          .K.......GP................V......NTGY.....M.V........
902           .K.......GP................V......NTGY.....M.VI..D.FLI
```

Fig. S11. Comparison of amino acid sequences of PBP1a mutants. The sequences shown correspond to the TP region from the Cp1015 (WT), Cba-19 and Cba-28 clinical strains, and the Cp1015 *pbp2b*^{19} (WT-1a^{19}, Cp1015 *pbp2b*^{28} (WT-1a^{28}) mutants. The R6 sequence is shown as a reference, and identical sites in other strains are represented by dots, with only polymorphisms being indicated. The amino acid position of the polymorphic sites are indicated vertically. Asterisks show the TP active sites. Amino acids in bold and underlined indicate the most common substitutions found in resistant isolates. Sequences are also compared to those described by Trzcinski *et al* (607T, 608, 901T and 908).

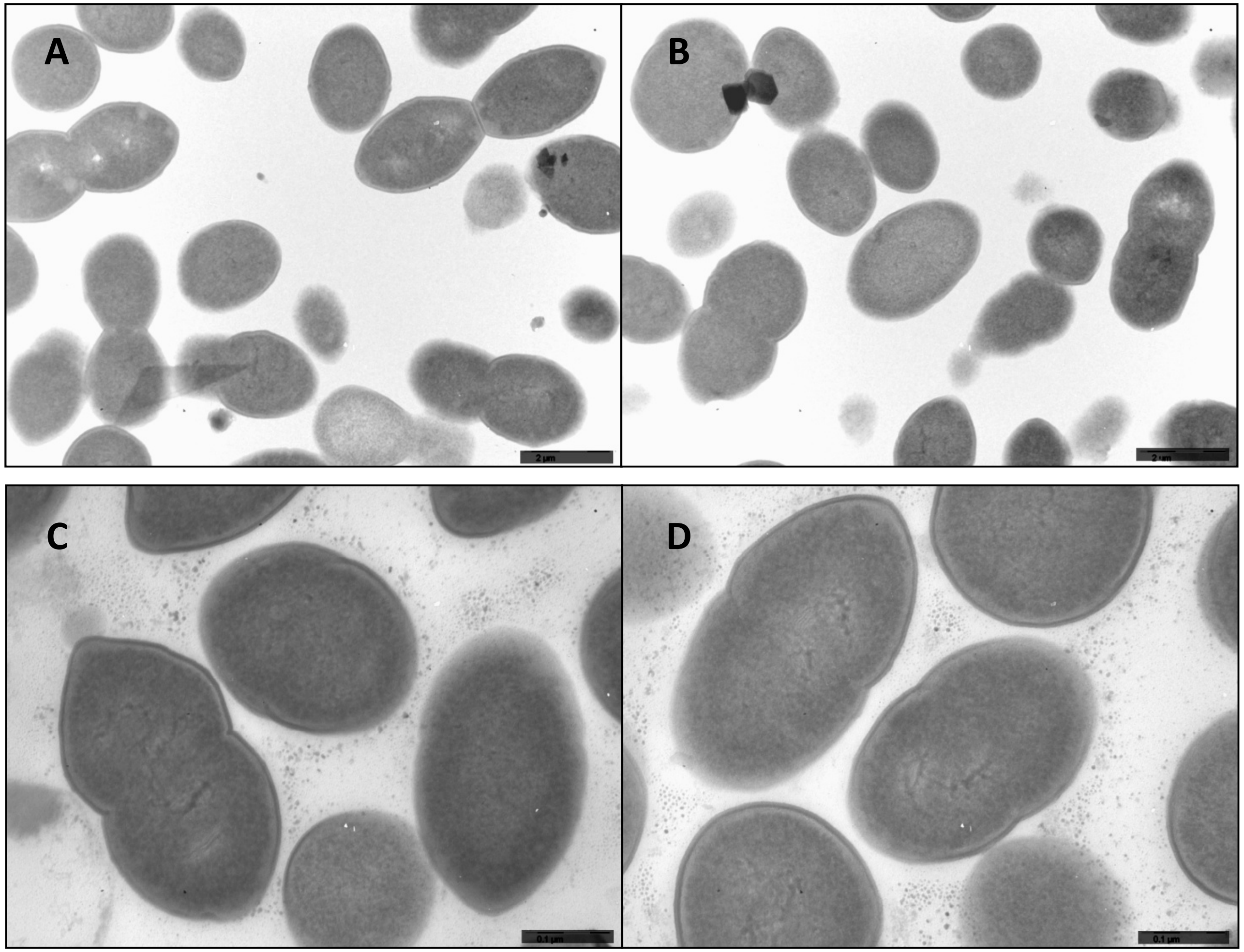

Fig. S12. Electron microscopy analysis of cellular morphology of strain Cp1015 (*wt)* Microphotographs of four different fields revealed the typical morphology of *S. pneumoniae*, with correct septum placement, division and symmetry of daughter cells. This data is complementary to that shown in Fig. 2 (A and B, bar scale 1.6 µm; C and D, bar scale 0.8 µm).

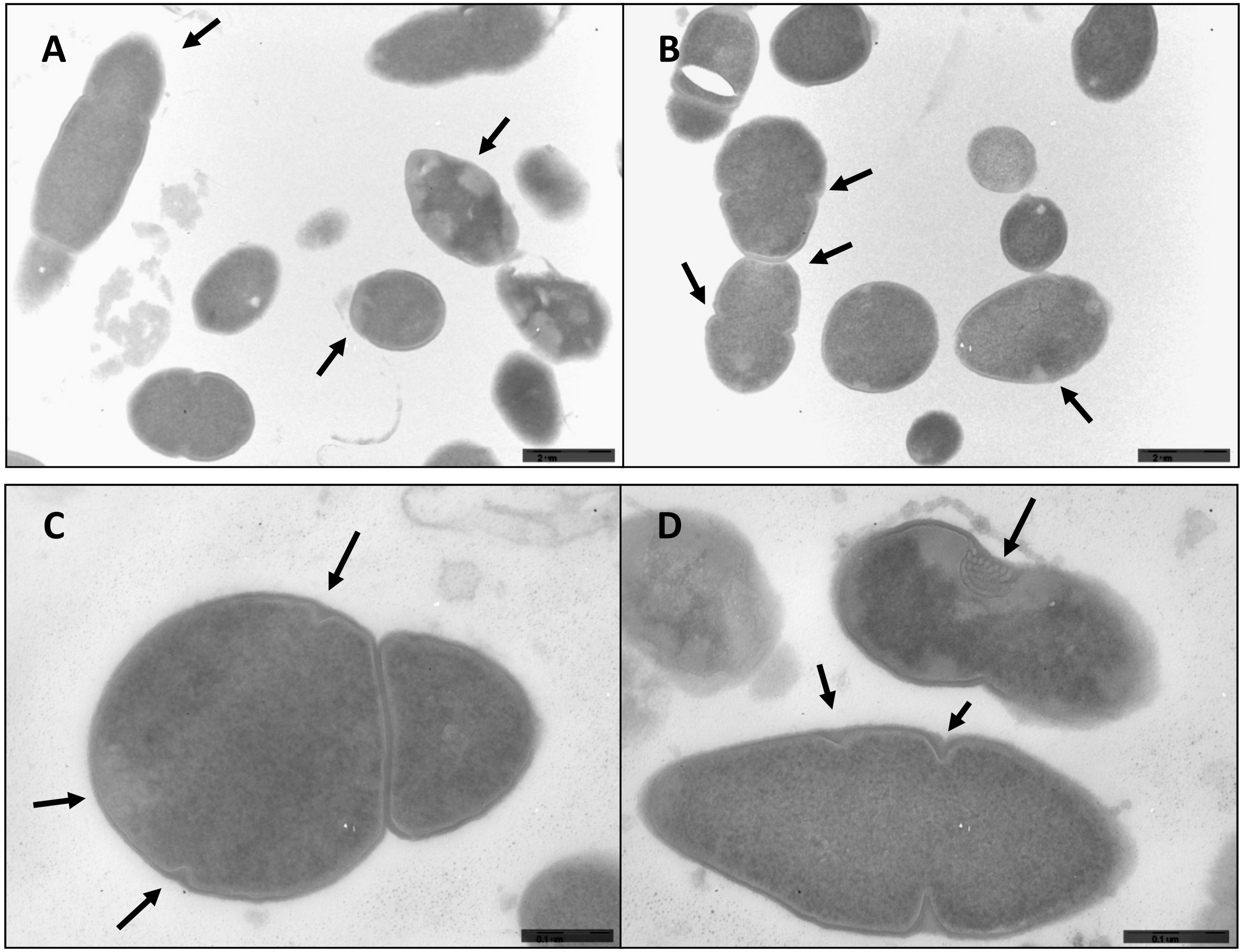

Fig. S13. Electron microscopy analysis of the effect of *pbp2b* mutations on cellular morphology. Microphotographs of different fields obtained for the *pbp2b* mutant. Arrows indicate multiple septa and irregularities, consistent with the accumulation of the cell wall, and atypical septum localization resulting in irregular cell divisions and asymmetrical daughter cells. This data is complementary to that shown in Fig. 2. (A and B, bar scale 1.6 µm; C and D, bar scale 0.8 µm).

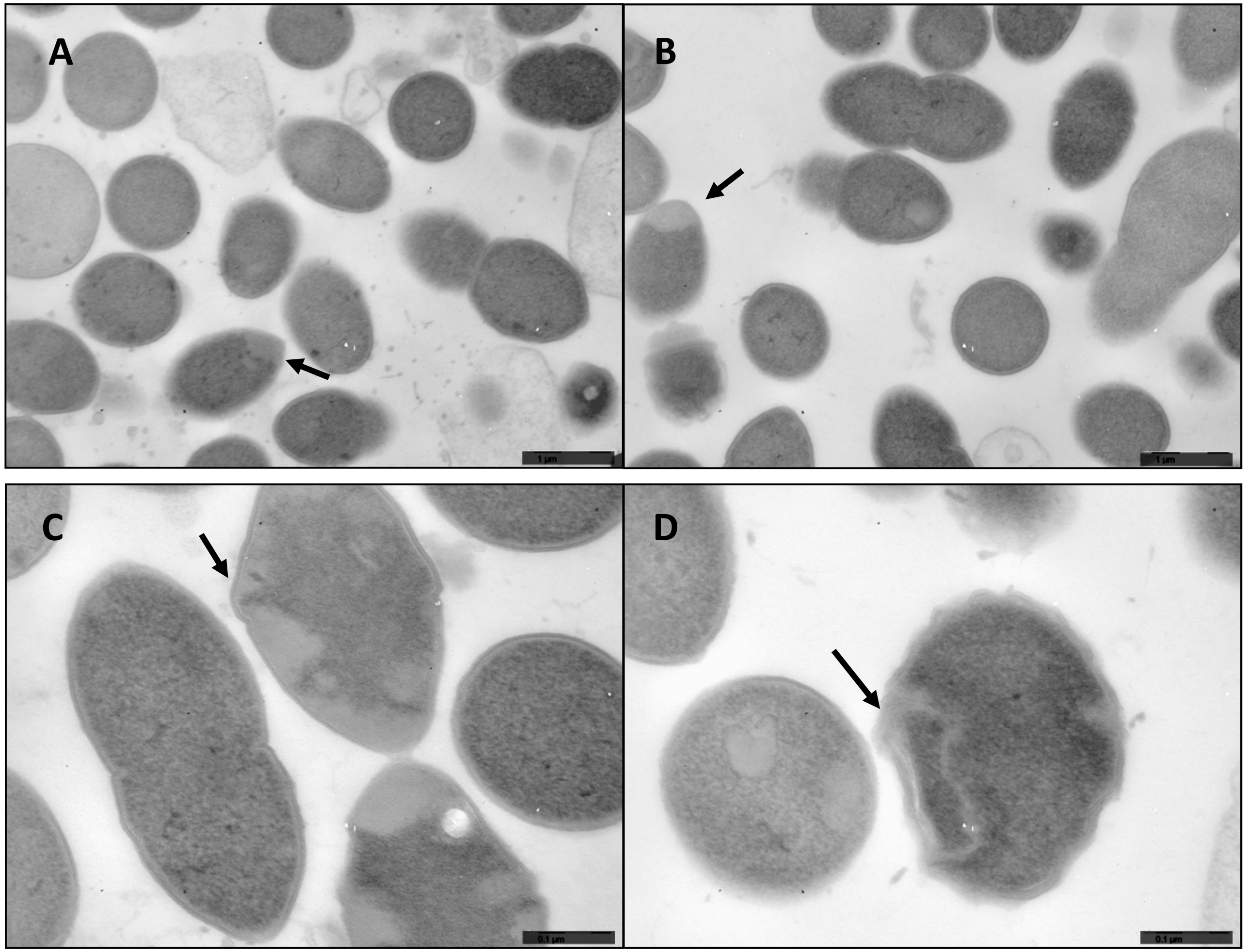

Fig. S14. Electron microscopy analysis of the effect of *pbp2b pbp2X* mutations on cellular morphology. Microphotographs of different fields obtained for the *pbp2b pbp2x* mutant. Arrows indicate irregularities consistent with the accumulation of the cell wall and atypical septum localization. This data is complementary to that shown in Fig. 2. (A and B, bar scale 1.6 µm; C and D, bar scale 0.8 µm).

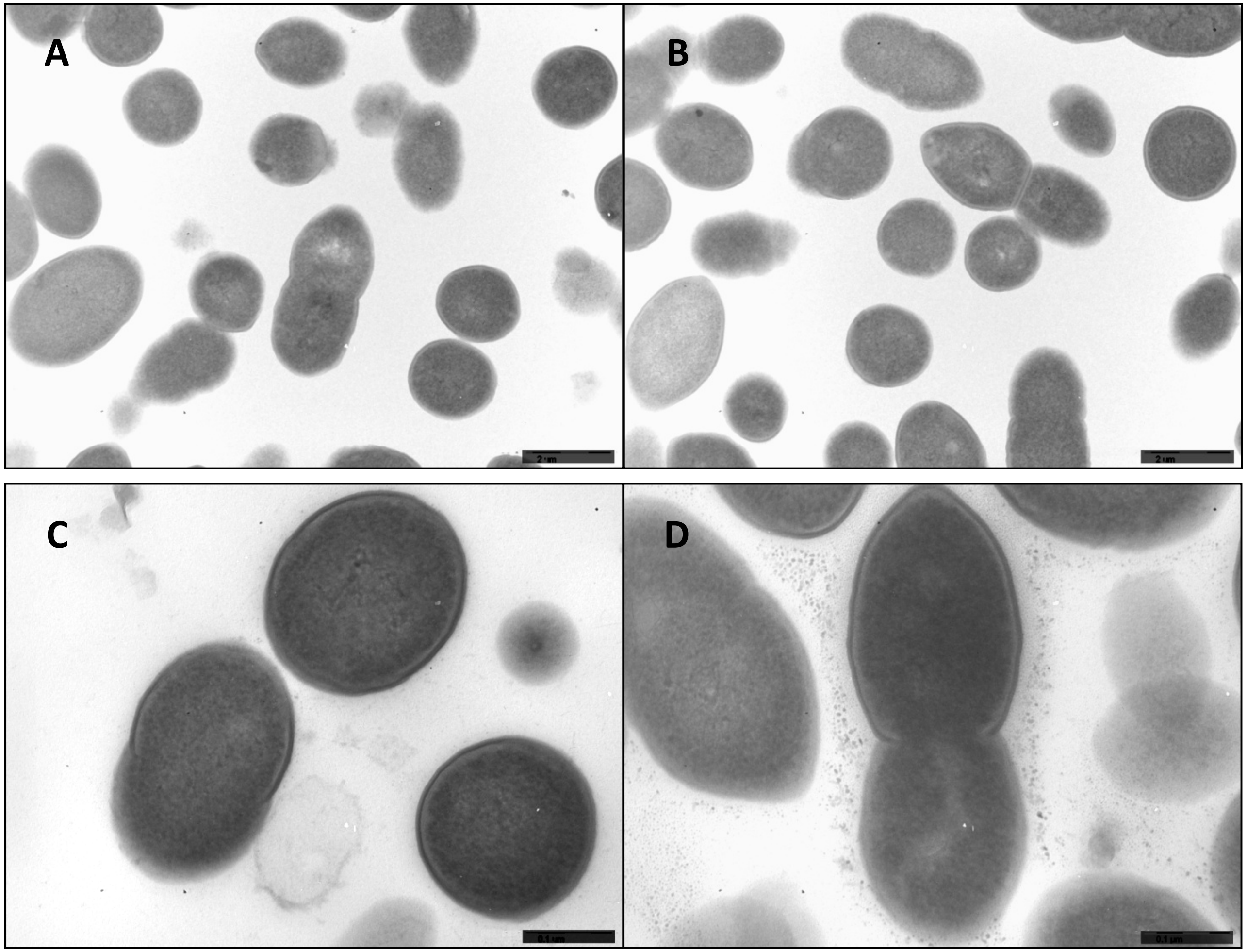

Fig. S15. Electron microscopy analysis of the effect of *pbp2b pbp2x pbp1a* mutations on cellular morphology. Microphotographs of different fields revealed no apparent abnormalities for the *pbp2b pbp2x pbp1a* mutant, which showed a similar morphology to that of wild-type cells. This data is complementary to that shown in Fig. 2. (A and B, bar scale 1.6 µm; C and D, bar scale 0.8 µm).

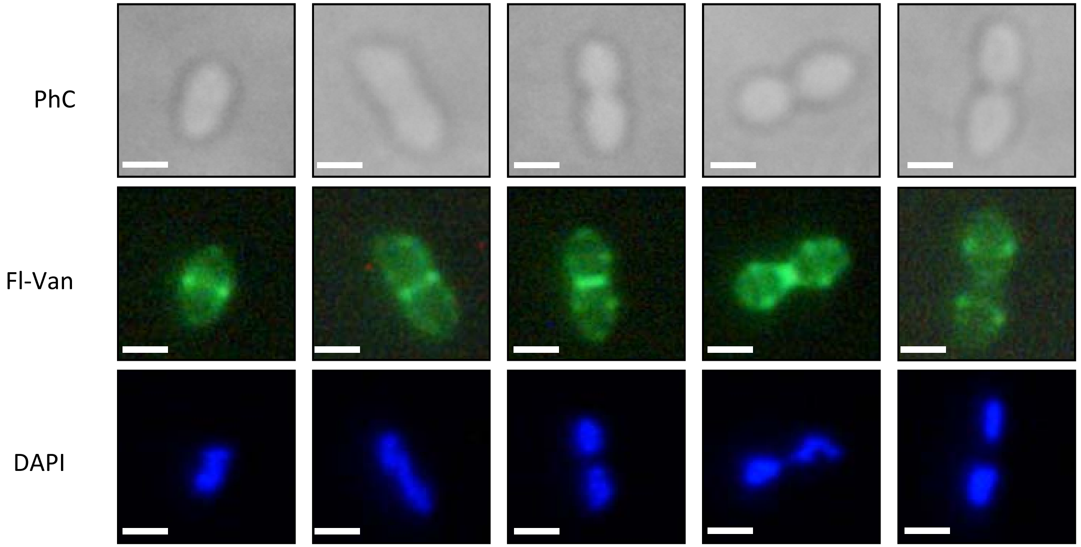

Fig. S16. Peptidoglycan synthesis of strain Cp1015 (*wt*) at different stages in the cell cycle. Cp1015 was grown exponentially in Todd Hewitt broth at 37ºC. Cells were stained with fluorescent vancomycin (Fl-Van) and DAPI, and viewed by using an epifluorescent microscope (see *Material and Methods*). Images were assigned to stages of the cell cycle on the basis of the extent of nucleoid segregation, cell elongation and septum constriction. Multiple fields were examined for numerous independent experiments, with representative images being shown (bar scale, 1 µm).

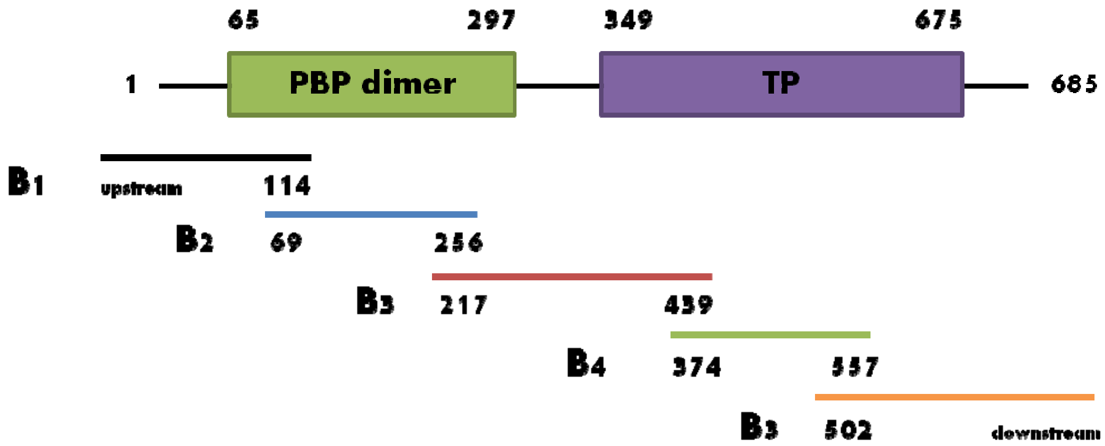

Fig. 17. Graphical representation of the *pbp2b* gene showing the different DNA fragments, amplified by PCR from clinical strains and sequenced (See Materials and Methods).
