# Supplementary material for: Compensatory Evolution of pbp Mutations Restores the Fitness Cost Imposed by β-Lactam Resistance in Streptococcus pneumoniae
Source: PLoS Pathog. 2011 Feb 17;7(2):e1002000. doi: 10.1371/journal.ppat.1002000 (PMC3040684; doi:10.1371/journal.ppat.1002000)
Supplement: Supporting Information S2 — Supplemental Tables S1 through S4. Table S1 Bacterial strains and plasmids used in this work. Table S2 Fitness cost analysis of double pbp2b pbp2x mutants obtained with pbp2x genes from different clinical strains. Table S3 Contribution of internal fragments of pbp1a and pbp2x in resistance and fitness compensation. Table S4 Primers used in this work. (PDF) [file ppat.1002000.s002.pdf]

Supporting Information-Tables

Table S1. Bacterial strains and plasmids used in this work

**Bacterial strains**	**Description**	**Source of reference**
Streptococcus pneumoniae		1
Cp1015	non-capsulated and βL susceptible laboratory strain	
D39	virulent βL-susceptible laboratory strain	NCTC 7466
R6	non-capsulated and βL susceptible laboratory strain	ATCC BAA-255
9V3	serotype-9V variant of the Spain^{9V}-3 international clone	ATCC 700671
Cba-12	βLR serotype-14 variant of the Spain^{9V}-3 clone	2
Cba-19	βLR serotype-14 variant of the Spain^{9V}-3 clone	2
Cba-28	βLR serotype-14 variant of the Spain^{9V}-3 clone	2
Cba-52	βLR serotype-14 variant of the Spain^{9V}-3 clone	2
Cba-54	βLR serotype-14 variant of the Spain^{9V}-3 clone	2
Cba-56	βLR serotype-14 variant of the Spain^{9V}-3 clone	2
Cba-62	βLR serotype-14 variant of the Spain^{9V}-3 clone	2
pbp2b^{19}	Cp1015 with *pbp2b* from Cba-19, PIP resistant	This work
pbp1a^{19}	Cp1015 with *pbp1a* from Cba-19, CTX resistant	This work
pbp2x^{19}	Cp1015 with *pbp2x* from Cba-19, CTX resistant	This work
pbp2b^{19}, *pbp1a*^{19}	Cp1015 with *pbp2b* and *pbp1a* from Cba-19, Pip and CTX resistant	This work
pbp2b^{19} *pbp2x*^{19}	Cp1015 with *pbp2b* and *pbp2x* from Cba-19, PIP and CTX resistant	This work
pbp2b^{19}*pbp2x*^{19} *pbp1a*^{19}	Cp1015 with *pbp2b*, *pbp1a* and *pbp2x* from Cba-19, PIP and CTX resistant	This work
pbp2b^{28}	Cp1015 with *pbp2b* from Cba-28 , PIP resistant	This work
pbp1a^{28}	Cp1015 with *pbp1a* from Cba-28, CTX resistant	This work
pbp2x^{28}	Cp1015 with *pbp2x* from Cba-28, CTX resistant	This work
pbp2b^{28} *pbp1a*^{28}	Cp1015 with *pbp2b* and *pbp1a* from Cba-28, PIP and CTX resistant	This work
pbp2b^{28} *pbp2x*^{28}	Cp1015 with *pbp2b* and *pbp2x* from Cba-28, PIP and CTX resistant	This work
pbp2b^{28} *pbp2x*^{28} *pbp1a*^{28}	Cp1015 with *pbp2b*, *pbp1a* and *pbp2x* from Cba-28, PIP and CTX resistant	This work
pbp2b^{9V3}	Cp1015 with *pbp2b* from 9V3, PIP resistant	This work
pbp2x^{9V3}	Cp1015 with *pbp2x* from 9V3, CTX resistant	This work
pbp2b^{9V3} *pbp2x*^{9V3}	Cp1015 with *pbp2b* and *pbp2x* from 9V3, Pip and CTX resistant	This work
pbp2b^{9V3} *pbp2x*^{9V3} pbp1a^{9V3}	Cp1015 with *pbp2b*, *pbp1a* and *pbp2x* from 9V-3, PIP and CTX resistant	This work
pbp2b^{28} *pbp2x*^{12}	Cp1015 with *pbp2b* from Cba-28, *pbp2x* from Cba-12, PIP and CTX resistant	This work
pbp2b^{28}*pbp2x*^{19}	Cp1015 with *pbp2b* from Cba-28, *pbp2x* from Cba-19, PIP and CTX resistant	This work
pbp2b^{28}*pbp2x*^{52}	Cp1015 with *pbp2b* from Cba-28, *pbp2x* from Cba-52, PIP and CTX resistant	This work
pbp2b^{28}*pbp2x*^{54}	Cp1015 with *pbp2b* from Cba-28, *pbp2x* from Cba-54, PIP and CTX resistant	This work
pbp2b^{28}*pbp2x*^{56}	Cp1015 with *pbp2b* from Cba-28, *pbp2x* from Cba-56, PIP and CTX resistant	This work
pbp2b^{28} *pbp2x*^{62}	Cp1015 with *pbp2b* from Cba-28, *pbp2x* from Cba-62, PIP and CTX resistant	This work
pbp2b^{28} *pbp2x*^{9V3}	Cp1015 with *pbp2b* from Cba-28, *pbp2x* from 9V-3, PIP and CTX resistant	This work
D39 *pbp2b*^{9V3}	D39 with *pbp2b* from 9V-3, virulent and PIP resistant	This work
D39 *pbp2b*^{9V3} *pbp2x*^{19}	D39 with *pbp2b* from 9V3 and *pbp2x* from Cba-19, virulent, PIP and CTX resistant	This work
D39 *pbp2b*^{9V3} *pbp2x*^{19} *pbp1a*^{19}	D39 with *pbp2b* from 9V-3 and *pbp2x* and *pbp1a* from Cba-19, virulent, PIP and CTX resistant	This work
pbp2b ^{28} *pbp1a* ^{28} A2	Cp1015 with *pbp2b* and an internal fragment (from 599 to 1002) of *pbp1a* from Cba-28 , PIP and CTX resistant	This work
pbp2b ^{28} *pbp1a* ^{28} A3	Cp1015 with *pbp2b* and an internal fragment (from 881 to 1294) of *pbp1a* from Cba-28 , PIP and CTX resistant	This work
pbp2b ^{28} *pbp2x* ^{28} X4	Cp1015 with *pbp2b* and an internal fragment (from 1286 to 2253) of *pbp2x* from Cba-28 , PIP and CTX resistant	This work
Cp1015 *pbp2bHA*::pEVP3::*pbp2b'*	CpCp1015 strain expressing PBP2b fused to the HA tag, CLM^{R}	This work
Cp1015 *pbp2xHA*::pEVP3::*pbp2x'*	Cp1015 strain expressing PBP2x fused to the HA tag, CLM^{R}	This work
Cp1015 *pbp1aHA*::pEVP3::*pbp1a'*	Cp1015 strain expressing PBP1a fused to the HA tag, CLM^{R}	This work
Cp1015 *pbp2b*^{28}*HA*::pEVP3::*pbp2b*^{28}'	Cp1015 *pbp2b*^{28} strain expressing PBP2b^{28} fused to the HA tag, CLM^{R}	This work
Cp1015 pbp2b^{28}	Cp1015 *pbp2b*^{28} strain expressing PBP2x fused to the HA tag, CLM^{R}	This work

pbp2xHA::pEVP3::*pbp2x'*		
Cp1015 pbp2b^{28} *pbp1aHA*::pEVP3::*pbp1a'*	Cp1015 *pbp2b*^{28} strain expressing PBP1a fused to the HA tag, CLM^{R}	This work
Cp1015 pbp2b^{28} *pbp2x*^{2} *pbp1a*^{28} *HA*::pEVP3::*pbp1a*^{28}*'*	Cp1015 triple *pbp* mutant expressing PBP1a^{28} fused to the HA tag, CLM^{R}	This work
Cp1015 pbp2b^{28} *pbp2x*^{28} *pbp1a*^{28} *HA*::pEVP3::*pbp2b*^{28}*'*	Cp1015 triple *pbp* mutant expressing PBP2b^{28} fused to the HA tag, CLM^{R}	This work
Cp1015 pbp2b^{28} *pbp2x*^{28} *pbp1a*^{28} *HA*::pEVP3::*pbp2x*^{28}*'*	Cp1015 triple *pbp* mutant expressing PBP2x^{28} fused to the HA tag, CLM^{R}	This work
Escherichia coli		
DH5α	used to propagate the pGEM-pbp plasmid	Invitrogen
Top10	used to propagate the pGFPTOPO plasmid	Invitrogen
XL1 Blue MR	used to co-transformed pBT and pTRG recombinant plasmids in order to test protein-protein interactions.	Stratagene
**Plasmids**		
pGEMT-easy	cloning vector	Promega
pCRTOPO 2.1	cloning vector	Invitrogen
pBT-LGF2	interaction control plasmid encoding the dimerization domain (40 amino acids) of the Gal4 transcriptional activator protein	
pTRG-Gal11	interaction control plasmid encoding a domain (90 amino acids) of the mutant form of the Gal11 protein.	Stratagene
pBT	contains a 3.2 kb-size bait plasmid that carries a low-copy p15A replication origin and confers chloramphenicol resistance. The plasmid encodes the full-length bacterial phage λ cI protein under the control of the IPTG-inducible lac-UV5 promoter.	Stratagene
pTRG	contains a 4.4 kb-size target plasmid that carries the low-copy ColE1 replication origin and confers tetracycline resistance. The plasmid directs transcription of the amino-terminal domain of RNA polymerase α subunit through a multiple cloning site at the 3´ end of the α subunit gene and is under the control of the IPTG-inducible, tandem promoter lpp/lac-UV5.	Stratagene
pGEM-pbp	contains the *pbp2b* coding sequence cloned into the pGEMT-easy vector	This work
pGFPTOPO	contains a *gfpmut3* copy under control of a pneumococcal promoter into the pCRTOPO 2.1 vector	This work
pBT-PBP2bwt	interaction plasmid encoding the full-length coding sequence of pbp2b gene obtained from Cp1015 wt strain	This work
pBT-PBP2b*	interaction plasmid encoding the full-length coding sequence of pbp2b gene obtained from Cba-28 clinical strain	This work
pTRG-ftsZ	interaction plasmid encoding the full-length coding sequence of ftsZ gene obtained from Cp1015 wt strain	This work
pTRG-PBP2xwt	interaction plasmid encoding the full-length coding sequence of pbp2x gene obtained from Cp1015 wt strain	This work
pTRG-PBP2x*:	interaction plasmid encoding the full-length coding sequence of pbp2x gene obtained from Cba-28 clinical strain	This work
pAT18	*E.coli-S.pneumoniae* shuttle vector, LacZα, SPC^{R}	3
pEVP3	*S. pneumoniae* insertion vector, CLM^{R}	4
pCM18	source of the *gfpmut3* gene	5
pAT18 *pbp2b*^{28}*-gfp*	pAT18 containing *pbp2b*^{28} fused to the *gfp* gene	This study
pAT18 *pbp-gfp*	pAT18 containing *pbp2b* fused to the *gfp* gene	This study
pEVP32bHA	pEVP3 containing the last 420 bp of *pbp2b* fused to the HA sequence tag	This study
pEVP32xHA	pEVP3 containing the last 342 bp of *pbp2x* fused to the HA sequence tag	This study
pEVP31aHA	pEVP3 containing the last 317 bp of *pbp1a* fused to the HA sequence tag	This study
pEVP32b^{28}HA	pEVP3 containing the last 420 bp of *pbp2b*^{28} fused to the HA sequence tag	This study
pEVP32x^{28}HA	pEVP3 containing the last 342 bp of *pbp2x*^{28} fused to the HA sequence tag	This study
pEVP31a^{28}HA	pEVP3 containing the last 317 bp of *pbp1a*^{28} fused to the HA sequence tag	This study

References: βLR, β-lactam resistant; CTX, cefotaxime; PIP, piperacillin; CLM: chloramphenicol; SPC, spectinomycin; Cba, Córdoba; 9V3, Spain^{9V}-3 clone.

1) Morrison, D. A., Lacks, S. A., Guild, W. R. and Hageman, J. M. (1983). Isolation and characterization of three new classes of transformation-deficient mutants of Streptococcus pneumoniae that are defective in DNA transport and genetic recombination. J. Bacteriol.156, 281-290.

2) Albarracin Orio AG, Cortes PR, Tregnaghi M, Pinas GE, & Echenique JR (2008) A new serotype 14 variant of the pneumococcal Spain9V-3 international clone detected in the central region of Argentina. J Med Microbiol 57(Pt 8):992-999.

3) Trieu-Cuot, P., et al., Shuttle vectors containing a multiple cloning site and a *lacZ* alpha gene for conjugal transfer of DNA from *Escherichia coli* to gram-positive bacteria. Gene, 1991. 102(1): p. 99-104.
4) Claverys JP, Dintilhac A, Pestova EV, Martin B, Morrison DA. 1995. Construction and evaluation of new drug-resistance cassettes for gene disruption mutagenesis in *Streptococcus pneumoniae*, using an *ami* test platform. Gene 164: 123-8
5) Hansen, M.C., et al., Assessment of GFP fluorescence in cells of *Streptococcus gordonii* under conditions of low pH and low oxygen concentration. Microbiology, 2001. 147(Pt 5): p. 1383-91.

Table S2. Fitness cost analysis of double *pbp2b pbp2x* mutants obtained by transformation of the pbp2b28 with different *pbp2x* alleles from clinical strains

pbp mutant	*pbp2x* donor clinical strain	cefotaxime MIC (µg/ml)	relative fitness in vitro[a,b]	SD	95% CI
pbp2b^{28}	28	0.015	0.65	0.02	0.62-0.68
pbp2b^{28} *pbp2x*^{12}	12	0,05	0,81	0,05	0.69-0.93
pbp2b^{28} *pbp2x*^{19}	19	0,15	0,86	0,05	0.74-0.98
pbp2b^{28} *pbp2x*^{28}	28	0,15	0,86	0.01	0.84-0.87
pbp2b^{28} *pbp2x*^{52}	52	0,08	0,64	0,05	0.52-0.76
pbp2b^{28} *pbp2x*^{54}	54	0,1	0,74	0,04	0.64-0.84
pbp2b^{28} *pbp2x*^{56}	56	0,05	0,72	0,05	0,60-0,84
pbp2b^{28} *pbp2x*^{62}	62	0,15	0,85	0,05	0.73-0.97
pbp2b^{28} *pbp2x*^{9V3}	Spain^{9V}-3	0,1	0,85	0,05	0.73-0.97

[a] Relative competitive fitness is given by the ratio of the number of generations of the resistant and susceptible CP1015 strains.
[b] Relative fitness in vitro was determined as described in Material and Methods.
References: 9V3; Spain^{9V}-3 international clone. CI, confidence intervals. SD: standard deviation

Table S3. Contribution of internal fragments of *pbp1a* and *pbp2x* to resistance and fitness compensation

pbp mutant	*pbp1a* fragment transformed	*pbp2x* fragment transformed	cefotaxime MIC (µg/ml)	relative fitness in vitro[b,c]	SD	95% CI
pbp2b^{28}	-	-	0,015	0.65	0.02	0.62-0.68
pbp2b^{28} *pbp1a*^{28}*A2*	599 to 1002	-	0,12	0,96	0,05	0,83-1.08
pbp2b^{28} *pbp1a*^{28}*A3*	881 to 1294	-	0,15	0,70	0,05	0,58-0.82
pbp2b^{28} *pbp2x*^{28}*X4*	-	1286 to 2253	0,15	0,64	0,05	0,52-0.76

Table S4. Primers used in this work

Primer Sequence	Target gene	Primer function
FX1 5'-CTTTCATAAGTATCTGGACATGG	*pbp2x*	Amplifying fragment X1 of *pbp2x* to transform Cp1015*pbp2b*[28]
RX1 5'-AGGACTGGAGGGGGCTGGAA	*pbp2x*	Amplifying fragment X1 of *pbp2x* to transform Cp1015*pbp2b*[28]
FX2 5'-AGGATCGTCTGGGTAATATTGT	*pbp2x*	Amplifying fragment X2 of *pbp2x* to transform Cp1015*pbp2b*[28]
Rx2 5'-GTCGCATCCGCTATTTTGAAT	*pbp2x*	Amplifying fragment X2 of *pbp2x* to transform Cp1015*pbp2b*[28]
Fx3 5'-GACTTTGTTTGGCGTGATATCC	*pbp2x*	Amplifying fragment X3 of *pbp2x* to transform Cp1015*pbp2b*[28]
Rx3 5'-GCAATAGCTGTAAAGGCACG	*pbp2x*	Amplifying fragment X3 of *pbp2x* to transform Cp1015*pbp2b*[28]
Fx4 5'-TGTTTATGATTCCACTGCAA	*pbp2x*	Amplifying fragment X4 of *pbp2x* to transform Cp1015*pbp2b*[28]
RX4 5'- CTTCTCTCGAGTTATTAGTCTCCTAAAGTTAAT GTAATTTTTTTA	*pbp2x*	Amplifying fragment X4 of *pbp2x* to transform Cp1015*pbp2b*[28]
Fa1 5'-GCTTCTTCGACCACAGGGGG	*pbp1a*	Amplifying fragment A1 of *pbp1a* to transform Cp1015*pbp2b*[28]
Ra1 5'-CAGATAAGACCAAGTTTCGG	*pbp1a*	Amplifying fragment A1 of *pbp1a* to transform Cp1015*pbp2b*[28]
Fa2 5'-TACCTCAGTTAGCCTTGCTG	*pbp1a*	Amplifying fragment A2 of *pbp1a* to transform Cp1015*pbp2b*[28]
Ra2 5'-TACGACCGTAGATGCGACTT	*pbp1a*	Amplifying fragment A2 of *pbp1a* to transform Cp1015*pbp2b*[28]
Fa3 5'-CTGGGATGGATGTTTACACA	*pbp1a*	Amplifying fragment A3 of *pbp1a* to transform Cp1015*pbp2b*[28]
Ra3 5'-TGACATTTCGTGATTGTTGA	*pbp1a*	Amplifying fragment A3 of *pbp1a* to transform Cp1015*pbp2b*[28]
Fa4 5'-TGTTTATGATTCCACTGCAA	*pbp1a*	Amplifying fragment A4 of *pbp1a* to transform Cp1015*pbp2b*[28]
Ra4 5'-CGTGTACCTACATCTGAAAATTC	*pbp1a*	Amplifying fragment A4 of *pbp1a* to transform Cp1015*pbp2b*[28]
Fa5 5'-TACGGAGCAAGTAGTGAAAAAAT	*pbp1a*	Amplifying fragment A5 of *pbp1a* to transform Cp1015*pbp2b*[28]
Ra5 5'-GCTTCCTTCAGACAGATACG	*pbp1a*	Amplifying fragment A5 of *pbp1a* to transform Cp1015*pbp2b*[28]
F2bdh 5'-CGGGATCCATGAGACTGATTTGTATGAGAA	*pbp2b*	Two hybrid system assay
R2bdh 5'-ATCACCTCGAGATTCATTGGATGGTATTTT	*pbp2b*	Two hybrid system assay
F2xdh 5'-GAGGATCTATGAAGTGGACAAAAAGAG	*pbp2x*	Two hybrid system assay
R2xdh 5'-GGATCTATGAAGTGGACAAAAAGAG	*pbp2x*	Two hybrid system assay
FftsZdh 5'-CGGGATCCATGACATTTTCATTTGATACAG	*ftsZ*	Two hybrid system assay
RftsZdh 5'-ATCACCTCGAGACGATTTTTGAAAAATGGAGGT	*ftsZ*	Two hybrid system assay
FB1 5'- TGCGATATCTGGTTTGTCCA	*pbp2b*	*pbp2b* gene sequencing
RB1 5'- TTGGAGAACTGATGCTCACA	*pbp2b*	*pbp2b* gene sequencing
FB2 5'- TGATGCTAGTGGAAAACCTT	*pbp2b*	*pbp2b* gene sequencing
RB2 5'- GGCTTCTGCTTCTTCCGCTG	*pbp2b*	*pbp2b* gene sequencing
FB3 5'- TGCCTGGCATTAGTATTTCT	*pbp2b*	*pbp2b* gene sequencing
RB3 5'- ATAGGGAATGAACCGTAAGC	*pbp2b*	*pbp2b* gene sequencing
FB4 5'- GTTGACGCCTGATTCCTTGG	*pbp2b*	*pbp2b* gene sequencing
RB4 5'- CCATAAATGCCTTCAACAAT	*pbp2b*	*pbp2b* gene sequencing
FB5 5'-TGAATCTACTGGATTTGTTCCC	*pbp2b*	*pbp2b* gene sequencing
RB5 5'- TCAAACGTCCACAAATAGAACAA	*pbp2b*	*pbp2b* gene sequencing

F2bf 5’-CTCGAGATGAGACTGATTTGTATGAGAA	*pbp2b*	*construction of pbp2b-GFP*
R2bf 5’-ATCACCTCGAGATTCATTGGATGGTATTTT	*pbp2b*	*construction of pbp2b-GFP*
Fgfp3 5’-CTATCCCGGGCCAAAATTTGTTTGATTTGTA TCTTAAAATTTTGTATAATAGGTAGAAAAGAGGAAGGAA ATAATAAATGGTCGACATGCGTAAAAGGAGAAGAACTTTTCAC	*gfp*	*construction of pbp2b-GFP*
Rgfp3 5’-CCTACCCGGGATTATTAAGGCCTTTTGTATAGTTCA TCCATGCCATG	*gfp*	*construction of pbp2b-GFP*
F2xtag 5`-CTAGTCTAGACTAGCCTATGCCCAGTGTCAAGGA	*pbp2x*	*construction of pbp2x-HA*
R2xHA 5-TATTATCCGCTCGAGTCAGTTAAGCATAATCTGGA ACATCGTAAGGATAGTCTCCTAAAGTTAATGTAATTTTTTTAATG	*pbp2x*	*construction of pbp2x-HA*
F1a-tag 5-CTAGTCTAGACTAGCGCTGCCAAAGTTTACCGCTCT	*pbp1a*	*construction of pbp1a-HA*
R1aHA 5- ATTATCCGCTCGAGTCAGTTAAGCATAATCTGG AACATCGTAAGGATATGGTTGTGCTGGTTGAGGATT	*pbp1a*	*construction of pbp1a-HA*
R2bHA 5-TATTATCCGCTCGAGTCAGTTAAGCATAATCTGGAA CATCGTAAGGATAATTCATTGGATGGTATTTTTGATACAG	*pbp2b*	*construction of pbp2b-HA*
F2btag 5-CTAGTCTAGACTAGTCGTGTGGCTCCTCGTATTG-3	*pbp2b*	*construction of pbp2b-HA*
